# Proteomic analysis of Nrf2 deficient transgenic mice reveals cellular defence and lipid metabolism as primary Nrf2-dependent pathways in the liver

**DOI:** 10.1016/j.jprot.2010.03.018

**Published:** 2010-06-16

**Authors:** Neil R. Kitteringham, Azman Abdullah, Joanne Walsh, Laura Randle, Rosalind E. Jenkins, Rowena Sison, Christopher E.P. Goldring, Helen Powell, Christopher Sanderson, Samantha Williams, Larry Higgins, Masayuki Yamamoto, John Hayes, B. Kevin Park

**Affiliations:** a MRC Centre for Drug Safety Science, School of Biomedical Sciences, University of Liverpool, Sherrington Buildings, Liverpool, Merseyside, L69 3GE, United Kingdom; b Department of Pharmacology, Faculty of Medicine, National University of Malaysia, Jalan Raja Muda Abdul Aziz, 50300 Kuala Lumpur, Malaysia; c AstraZeneca R&D Alderley Park, Safety Assessment UK, Mereside, Alderley Park, Macclesfield, Cheshire, SK10 4TG England; d Biomedical Research Centre, Ninewells Hospital and Medical School, University of Dundee, Dundee DD1 9SY, Scotland, United Kingdom; e Division of Medical Biochemistry, Tohoku University Graduate School of Medicine, Tohoku, Japan

**Keywords:** Nrf2, Transgenic, Liver, Protein expression, iTRAQ, Lipid metabolism

## Abstract

The transcription factor Nrf2 regulates expression of multiple cellular defence proteins through the antioxidant response element (ARE). Nrf2-deficient mice (Nrf2^−^^/^^−^) are highly susceptible to xenobiotic-mediated toxicity, but the precise molecular basis of enhanced toxicity is unknown. Oligonucleotide array studies suggest that a wide range of gene products is altered constitutively, however no equivalent proteomics analyses have been conducted. To define the range of Nrf2-regulated proteins at the constitutive level, protein expression profiling of livers from Nrf2^−/−^ and wild type mice was conducted using both stable isotope labelling (iTRAQ) and gel electrophoresis methods. To establish a robust reproducible list of Nrf2-dependent proteins, three independent groups of mice were analysed. Correlative network analysis (MetaCore) identified two predominant groups of Nrf2-regulated proteins. As expected, one group comprised proteins involved in phase II drug metabolism, which were down-regulated in the absence of Nrf2. Surprisingly, the most profound changes were observed amongst proteins involved in the synthesis and metabolism of fatty acids and other lipids. Importantly, we show here for the first time, that the enzyme ATP-citrate lyase, responsible for acetyl-CoA production, is negatively regulated by Nrf2. This latter finding suggests that Nrf2 is a major regulator of cellular lipid disposition in the liver.

## Graphical abstract

**Figure:**
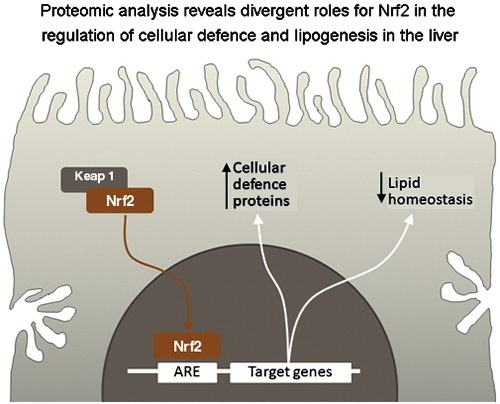

## 1 Introduction

Exposure to electrophiles and reactive oxygen species (ROS) may result in intracellular damage to proteins, DNA and other macromolecules and can lead to the development of diseases, such as cancer, neurodegenerative disorders and cardiovascular disease [1–3]. To counteract the damage caused by electrophiles and ROS, higher animals have developed elaborate defence mechanisms [4,5], which include the coordinated induction of a battery of genes encoding phase II detoxifying enzymes and oxidative stress inducible proteins [6,7]. It is now well established that a principal regulator of the cellular defence response is the transcription factor termed *nuclear factor erythroid-2 related factor 2* (Nrf2) [8–15]. Nrf2 been shown to play a key role in the transcriptional activation of multiple genes involved in cellular defence against ROS and electrophiles, such as NAD(P)H:quinone oxidoreductase (NQO1) [16], glutathione S-transferases (GSTs) [17,18], glutamate-cysteine ligase [19], haem oxygenase-1 (HO-1) [20], thioredoxin [12], and ferritin [21].

Nrf2 deficient transgenic mice have provided the most informative integrated model in which to examine the role of Nrf2 in regulating the defence response to chemical insults, particularly in the liver. Inactivation of the *nrf2* gene results in no obvious phenotypic changes (except in aging female animals, where autoimmune diseases have been observed [22]), indicating that Nrf2 is not essential for normal growth and development [23]. Several studies focussing on individual proteins have shown that the presence of Nrf2 is essential for the *enhanced* expression of several antioxidant response proteins following administration of certain chemical inducers, however, *constitutive* expression of the same genes is often unaffected or only marginally reduced by deletion of the Nrf2 gene [9,11,14,23–29].

Acute exposure of Nrf2-deficient mice to a range of toxic chemical insults has been shown to result in enhanced toxicity compared to their wild type counterparts. Multiple studies show a reduced resistance to hepatotoxicity induced by a wide range of compounds, including paracetamol [25,30], carbon tetrachloride [31], pyrazole [32], ethanol [33] and pentachlorophenol [34].

Two possible explanations exist for the reduction in chemically-induced hepatotoxicity seen in each of these studies: first, the lack of Nrf2 may abrogate the animal**'**s ability to up-regulate defence proteins in response to the chemical stimulus or, second, the enhanced toxicity may simply reflect a constitutive reduction in defence proteins due to the absence of Nrf2. Clearly, these two possible mechanisms are not mutually exclusive and each may contribute to a different degree, depending on the nature of the chemical insult. Nevertheless, it is important to understand the relative contribution of each mechanism for a given hepatotoxin in order to translate information gained in animal studies into improved clinical management of drug- or chemical-induced toxicity in man.

Oligonucleotide microarray analysis of Nrf2 null mice suggests that Nrf2 may regulate more than 200 genes, either constitutively or following exposure to a known inducer [29,35,36]. However, it is now well recognized that transcriptional up-regulation does not always equate to increased protein expression [37]. Until now, no equivalent proteomic analysis of Nrf2 null mice has been undertaken to substantiate the mRNA expression changes at the protein level. Here we report a global analysis of constitutive hepatic protein expression in Nrf2 null and wild type mice [10].

## 2 Materials and methods

### 2.1 Materials

Protein assay kits were from Bio-Rad (Hemel Hempstead, Herts, UK). Immobiline Dry Strips and associated buffers for 2-DE gels were obtained from GE Healthcare UK (Little Chalfont, Bucks, UK). 8-plex isobaric tags for relative and absolute quantification (iTRAQ) protein labelling kit/reagents were purchased from AB Sciex (Framingham, MA, USA). Sequencing grade trypsin was obtained from Promega UK (Southampton, Hants, UK). All other reagents were obtained from Sigma (Poole, Dorset, UK).

### 2.2 Animal studies

All experiments were undertaken in accordance with criteria outlined in a license granted under the Animals (Scientific Procedures) Act 1986, and approved by the Animal Ethics Committees of the University of Liverpool. Generation of the Nrf2 knockout mouse and genotyping of progeny have been described elsewhere [26,28]. Male mice of approximately 10 weeks of age were used throughout the study. Mice were housed at a temperature range of 19 °C–23 °C under 12-h light/dark cycles and given free access to food and water. Animals were killed by exposure to a rising concentration of CO_2_ followed by cervical dislocation. Livers were removed and snap-frozen immediately in liquid N_2_, before being stored at −80 °C.

Three groups of mice were used: for the first iTRAQ analysis (*iTRAQ analysis 1*), 8 mice (4 Nrf^(+/+)^ and 4 Nrf^(^^−^^/^^−^^)^) were used, for the second iTRAQ analysis (*iTRAQ analysis 2*), 12 mice (6 Nrf^(+/+)^ and 6 Nrf^(^^−^^/^^−^^)^) and for the 2DE gel analysis 8 mice were used (4 Nrf^(+/+)^ and 4 Nrf^(^^−^^/^^−^^)^).

### 2.3 iTRAQ labelling of liver homogenates

Whole liver homogenates (75 μg protein) from Nrf2^(+/+)^ and Nrf2^(^^−^^/^^−^^)^ (*n* = 4), were prepared in TEAB/SDS. iTRAQ reagent labelling was then carried out according to the Applied Biosystems protocol for an 8plex procedure. Briefly, samples were denatured, reduced and capped with methylmethanethiosulfate (MMTS), before overnight digestion with trypsin and then labelled with isobaric tags. For the first three iTRAQ runs, Nrf2^(+/+)^ samples were labelled with tags 113 to 116 while Nrf2^(^^−^^/^^−^^)^ samples received the 117 to 121 tags. In the fourth experiment, the sample labelling was reversed such that the wild type animals had the heavier tags and the null mice the lighter tags, in order to control for labelling bias. iTRAQ-labelled peptides were then pooled and diluted to 4 mL with 10 mM potassium dihydrogen phosphate/25% acetonitrile (ACN; w/v). The pH of the samples was adjusted to < 3 using phosphoric acid prior to fractionation on a Polysulfoethyl A strong cation-exchange column (200 × 4.6 mm, 5 μm, 300 Å; Poly LC, Columbia, MD). A flow rate of 1 mL/min was applied and peptides eluted by increasing the concentration of KCl in the mobile phase to 0.5 M over 60 min. Fractions of 2 mL were collected and were dried by centrifugation under vacuum (SpeedVac, Eppendorf).

### 2.4 Mass spectrometric analysis of iTRAQ samples

For LC-MS/MS analysis of iTRAQ labelled samples, each cation exchange fraction was resuspended in 120 μL 5% ACN/0.05% trifluoroacetic acid (TFA) and 60 μL were loaded on column. Samples were analysed on a QSTAR® Pulsar i hybrid mass spectrometer (AB Sciex) and were delivered into the instrument by automated in-line liquid chromatography (integrated LCPackings System, 5 mm C18 nano-precolumn and 75 μm × 15 cm C18 PepMap column; Dionex, California, USA) via a nano-electrospray source head and 10 μm inner diameter PicoTip (New Objective, Massachusetts, USA). The precolumn was washed for 30 min at 30 μL/min with 5% ACN/0.05% TFA prior to initiation of the solvent gradient in order to reduce the level of salt in the sample. A gradient from 5% ACN/0.05% TFA (v/v) to 60% ACN/0.05% TFA (v/v) in 70 min was applied at a flow rate of 300 nL/min. The MS was operated in positive ion mode with survey scans of 1 s, and with an MS/MS accumulation time of 1 s for the three most intense ions. Collision energies were calculated on the fly based on the *m/z* of the target ion and the formula, collision energy = (slope × *m/z*) + intercept. The intercepts were increased by 3–5 V compared to standard data acquisition in order to improve the reporter ion intensities/quantitative reproducibility.

### 2.5 iTRAQ data analysis

Data analysis was performed using ProteinPilot software (Version 3, AB Sciex, Warrington, UK). The data were analysed with a fixed modification of MMTS-labelled cysteine, biological modifications allowed and with the confidence set to 10% to enable the False Discovery Rate to be calculated from screening the reversed SwissProt database. Ratios for each iTRAQ label were obtained, using a wild type mouse (WT mouse 1) sample as the denominator. The detected protein threshold (“unused protscore (conf)”) in the software was set to 1.3 to achieve 95% confidence.

### 2.6 Network analysis

The accession numbers of the 108 proteins identified as significantly different following Benjamini–Hochberg adjustment for multiple comparisons (*p* ≤ 0.2) were converted to Entrez gene IDs using the *D*atabase for *A*nnotation, *V*isualization and *I*ntegrated *D*iscovery (DAVID) (http://david.abcc.ncifcrf.gov/conversion.jsp ) and analysed for evidence of network wide changes in cellular phenotype using MetaCore from GeneGo Inc., an integrated manually curated knowledge database for pathway analysis of gene lists (http://www.genego.com/metacore.php).

The gene list was analysed using the Pathway Maps tool, which maps the genes listed to defined signalling pathways that have been experimentally validated and are widely accepted. The proteins deemed Nrf2-regulated according to the criteria defined above were compared against a background file containing all of the identified proteins which had similarly been converted to a list of Entrez gene IDs using DAVID. The *p* values generated by the software were used to determine the statistical significance of the pathways identified. The *p* value represents the probability that a particular pathway will be represented by chance given the number of genes in the experiment and the number of genes in the pathway.

### 2.7 2-DE of liver homogenates

Mouse livers were weighed and 0.3 g of tissue was homogenized in 1 mL lysis buffer [40 mM tris, 7 M urea, 2 M thiourea, 4% (w/v) 3-[(3-Cholamidopropyl)-dimethylammonio]-1-propane sulfonate (CHAPS), 10 mM 1,4-dithiothreitol (DTT), 1 mM EDTA]. The homogenate was sonicated for 30 s and centrifuged at 150 000 *g* for 45 min. The supernatant was assayed for protein content [38] and stored at −80 °C. Aliquots of samples containing equal quantities of protein (0.5 mg) were diluted to 350 μL with rehydration buffer (9 M urea, 2% w/v CHAPS, bromophenol blue (trace), 2% v/v immobilized pH gradient (IPG) buffer, 0.28% w/v DTT) and incubated overnight with nonlinear Immobiline DryStrips (18 cm; pH 3–10 non-linear) in a re-swelling chamber. The samples were separated in the 1st dimension by isoelectric focussing (IEF) for 25 h at a constant temperature of 20 °C to achieve a total of 75 000 Vh (MultiPhor II, GE Healthcare UK, Little Chalfont, Bucks, UK). The IPG strips were then incubated with equilibration buffer (50 mM Tris, 6 M urea, 30% v/v glycerol, 2% w/v SDS, bromophenol blue (trace) containing 1% w/v DTT) for 15 min followed by incubation in the same buffer with the DTT replaced by 2.5% w/v iodoacetamide for a further 15 min. The strips were applied to the surface of 12% w/v SDS-PAGE gels and sealed with agarose [39]. The samples were subjected to electrophoresis at 20 W/gel and 25 °C for approximately 3 h (Ettan Dalt 12, GE Healthcare UK, Little Chalfont, Bucks, UK). The gels were then stained with colloidal Coomassie blue.

### 2.8 Image analysis of 2-DE gels

Colloidal Coomassie blue stained 2D gels were scanned using a GS710 calibrated imaging densitometer (BioRad, Hemel Hempstead, UK). TIFF images were generated and were analysed using ImageMaster^TM^ 2D Elite software, version 4.01 (Amersham Pharmacia Biotech, Buckinghamshire, England). Altogether eight gels were analyzed (4 Nrf2^(^^−^^/^^−^^)^ and 4 Nrf2^(+/+)^). An objective strategy for quantitative comparisons between wild type and null liver samples was adopted to exclude the possibility of bias, as follows. The gels were initially analysed using an automated procedure to identify spots. The authenticity and outline of each spot was validated by eye and edited where necessary. In each case approximately 500 validated spots were recorded from each gel. Spot matching was accomplished initially by automated fitting of the spots, followed by manual seeding of remaining spots that failed to match by automated fitting. A background value was subtracted for each gel and the spot volumes normalised against the total volume of all matched spots. For each spot, the ratio between its intensity and the sum of all spot intensities in the gel (normalized spot intensity) was determined and used for quantitative comparison. Visual and quantitative comparisons were only sought in spots that were matched in all four gels for a given treatment group.

### 2.9 Identification of proteins from 2DE gels

Protein spots of interest were excised from Colloidal Coomassie blue-stained 2DE gels by automated spot excision (Ettan Dalt Spot Picker, Amersham Biosciences) and were subjected to tryptic digestion [40]. Gel pieces were washed with 100 μl of 50% (v/v) ACN/50 mM ammonium bicarbonate (NH_4_CO_3_) (pH 7.8) for 15 min and were dried by centrifugation under vacuum (SpeedVac, Eppendorf). The dried gel pieces were rehydrated with 4–10 μl of digestion buffer (5 ng/μl of modified sequencing grade trypsin in 50 mM NH_4_CO_3_) and were incubated overnight at 37 °C. The resulting peptides were extracted by the addition of 30 μl of 60% ACN/1% TFA and incubation for 5 min in a sonicating water bath at 20 °C. The samples were briefly centrifuged and the supernatants were collected. A further 30 μl of 60% ACN/1% TFA was added to the gel plug and the sample was sonicated for 5 min. The supernatants were pooled and dried by centrifugation under vacuum. The peptides were resuspended in 10 μl of 5% ACN/0.05% TFA. 0.5 μl of the peptide mixture was spotted onto a 96-position stainless steel target and was mixed 1:1 with matrix [10 mg/ml α-cyano-4-hydroxycinnamic acid in 50% ACN/ 0.1%TFA]. Peptide mass finger prints were obtained on a Voyager DE Pro MALDI mass spectrometer (AB Sciex) and the resulting mass lists searched against the NCBInr database using Mascot software (Matrix Science). Scores of greater than 75 were regarded as sufficient for identification. Each significant identification was checked for consistency between its isoelectric point, molecular mass and mobility on the 2DE gel and, where possible, coincidence with a published mouse liver proteome 2DE database [41,42].

### 2.10 Western immunoblotting for ATP-citrate lyase

Whole liver homogenate (25 µg of protein) was separated by denaturing electrophoresis on a 10% polyacrylamide gel (ProtoGel acrylamide solution and buffers, using Tris-Glycine-SDS running buffer) and transferred to a nitrocellulose membrane (GE healthcare). After transfer, a Ponceau Red stain was used to ensure equal loading and then the membrane was blocked using 10% milk in 1x TBS/0.1%Tween for 30 min at room temperature, before incubation with a rabbit monoclonal antibody to ATP citrate lyase (ab40793, Abcam plc, Cambridge, UK) at 1:2000 with 2% milk in 1xTBS/0.1%Tween at 4 °C overnight. The membrane was washed several times with TBS-Tween and then incubated with the secondary antibody (peroxidise-conjugated goat anti-rabbit immunoglobulin G, 1:10 000 in TBS-Tween containing 2% milk) for 1 h at room temperature. Enhanced Chemiluminescence Plus (GE Healthcare) was used to visualise the level of protein-antibody complex. Band volume was measured by densitometry using Biorad Quantity One 1D Analysis Software (BioRad).

### 2.11 Identification of antioxidant response elements in the promoter regions of Nrf2-regulated genes

ARE consensus sequences were sought in the 5′-flanking regions upstream of all genes identified in the initial iTRAQ analysis (iTRAQ analysis 1) as being Nrf2-regulated (*p* < 0.05, Student's *t*-test). Public domain software (Regulatory Sequence Analysis Tools**,** http://rsat.ulb.ac.be/rsat/) provided by the *Service de Conformation des Macromolécules Biologiques et de Bioinformatique* at the University Libre de Bruxelles [43] was used. 5′-flanking sequences (2000 bp upstream of the start codon) were retrieved directly from the *ENSEMBL* database from within the RSAT package. Promoter sequences were then interrogated for ARE or ARE-like sequences using both string-based and matrix-based protocols. String-based analysis was carried out using the programme ‘*dna search*’ available within the RSAT web resource. The search term used was RTGABNNNGCA (where R = G/C, B = G/C/T and N = any nucleotide) based on the consensus sequence derived by Nioi et al. [44]. In order to identify ARE-like sequences, a matrix-based pattern matching method was performed using the programme ‘*patser’*. A position-specific scoring matrix (PSSM) was created based on the core ARE (cARE) position-specific probability matrix published by Nerland [45]. In order to calculate the background base frequencies, A/T and C/G frequencies were determined within the upstream sequences of all the genes interrogated and a mean value for each base was defined (A/T 0.26, C/G 0.24). The derived PSSM was then used to scan each of the genes shown to be significantly different between Nrf2^(+/+)^ and Nrf2^(^^−^^/^^−^^)^ mice.

### 2.12 Statistical analysis

#### 2.12.1 iTRAQ data

iTRAQ data for proteins within the 1% false discovery rate and for which full quantification data were obtained, were statistically analysed within the R computational environment [46]. R is an open source software environment for statistical computing and graphics (http://www.r-project.org/). Normality of data and equivalence of variance across the data sets was assessed by Shapiro–Wilk and *F*-tests, respectively, and also by inspection of histogram plots for all proteins identified. Data were then analysed by *t*-test using the module *multtest*, a package designed for re-sampling based multiple hypothesis testing. Benjamini–Hochberg corrections for multiple comparisons were performed on all raw *p* values generated [47]. Protein expression differences between wild type and Nrf2-null mice giving a *p* value of < 0.05 by *t*-test and a Benjamini–Hochberg value ≤ 0.2 were accepted for further correlative network analysis. The Benjamini–Hochberg cut-off was set at 0.2 to avoid the exclusion of correlated Nrf2-regulated proteins through application of too stringent a correction for multiple testing in accordance with multivariate modelling approaches to account for potential confounders [47].

#### 2.12.2 2DE gel data

Data are expressed as mean ± SEM for at least four separate experiments. All values were analysed for non-normality using the Shapiro–Wilk test. Normally distributed values were compared using Student's unpaired *t*-test whilst non-normal values were analysed using the Mann–Whitney test. These statistical analyses were performed using the SPSS statistical software package, version 12 (Chicago, IL, USA). Statistical significance was accepted at *p* values of < 0.05.

## 3 Results

### 3.1 iTRAQ analysis of Nrf2^(+/+)^ and Nrf2^(^^−^^/^^−^^)^ mouse liver proteins

Two independent sets of mice were analysed using iTRAQ stable isotope labelling. For the first analysis (iTRAQ analysis 1), samples from four Nrf2^(+/+)^ and four Nrf2^(^^−^^/^^−^^)^ mice were analysed using 8-plex iTRAQ reagents and the entire analysis was repeated on four separate occasions. For the second group of mice (iTRAQ analysis 2), six Nrf2^(+/+)^ and six Nrf2^(^^−^^/^^−^^)^ mice were compared on a single occasion using three sets of 4-plex iTRAQ reagents. The second set of mice was thus used as a validation cohort to challenge the reproducibility of the protein changes observed in the initial “training” set. In each case iTRAQ data were processed using Protein Pilot version 3, including FDR. Table 1 shows the numbers of proteins identified and quantified within the two independent iTRAQ analyses.

#### 3.1.1 iTRAQ analysis 1

**Fig. 1:**
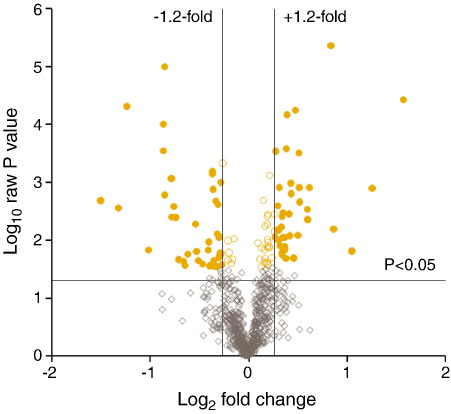
Volcano plot of the entire set of proteins quantified during iTRAQ analysis 1. Each point represents the difference in expression (fold-change) between Nrf2^(+/+)^ and Nrf2^(−/−)^ mice plotted against the level of statistical significance. Solid lines represent differential expression differences of ± 20% and a significance level of *p* < 0.05 (Student's *t*-test). Proteins represented by diamonds were not differentially expressed. Circles represent proteins that gave a raw *p* value of < 0.05 and Benjamini–Hochberg value of ≤ 0.2.

For iTRAQ analysis 1, each of the four runs represents a full repeat analysis of the same mouse liver sample. Thus, each protein expression value derived from iTRAQ analysis 1 represents the mean from four animals repeated on four occasions. In total, 1109 unique proteins were identified in at least one of the four runs within the FDR of 1% (Table 1); of these, 769 proteins had complete data sets in at least one run across all eight mice, and were consequently accepted for full quantitative analysis. Considerable variation was seen between the four runs, with the first run in particular giving relatively low proteome coverage. Nevertheless, all four runs were included for statistical analysis in order to maximise the number of proteins to include in the network analysis. Following statistical analysis, 108 proteins were found to be differentially expressed between Nrf2^(+/+)^ and Nrf2^(^^−^^/^^−^^)^ mouse livers using the criteria defined above and these are listed in Table 2. Fig. 1 shows a volcano plot of the entire data set highlighting proteins whose expression was significantly different (*t*-test *p* < 0.05) between wild type and Nrf2-null mice (open circles). Proteins that were significantly different by at least 20% are shown as filled circles. Approximately equivalent numbers of proteins were found to be up-regulated in Nrf2^(^^−^^/^^−^^)^ mice as were down-regulated. Whilst those that were significantly less abundant in Nrf2 null animals corresponded mainly to proteins involved in phase II and phase III drug disposition, in line with previous oligonucleotide array and immunoblotting experiments, those that were up-regulated were mostly involved with lipid metabolism. Proteins whose function is identified within the Uniprot database (http://www.uniprot.org/) as lipid metabolism or lipid transport are summarized in Table 3.

#### 3.1.2 iTRAQ analysis 2

For iTRAQ analysis 2, each run represents a single analysis of a different set of wild-type and Nrf2-null liver samples. In this case, 1070 proteins were initially identified with a FDR below 1%, of which 628 were associated with full quantitative datasets (Table 1). There was little variation between the three runs with respect to protein numbers identified, however the final number of unique proteins quantified was slightly lower than in iTRAQ analysis 1. Following Student's *t*-test analysis with adjustment for multiple testing by Benjamini–Hochberg analysis, thirty eight proteins were found to be differentially regulated between Nrf2^(+/+)^ and Nrf2^(^^−^^/^^−^^)^ liver samples, as shown in Table 4.

#### 3.1.3 Cellular defence and lipid metabolism are the primary biochemical functions regulated by Nrf2

**Fig. 2:**
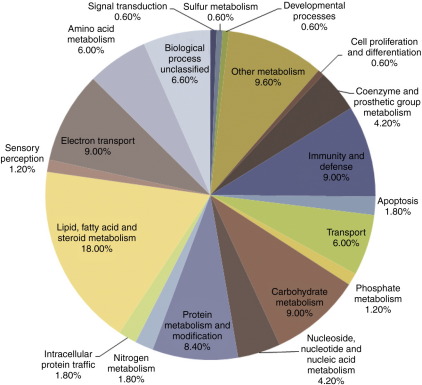
Panther functional classification of proteins shown to be differentially regulated in the Nrf2^(−/−)^mouse model.

The proteins identified as Nrf2-regulated by the two iTRAQ analyses were independently subjected to correlative network analysis. The 108 proteins obtained from the initial iTRAQ analysis were submitted to the PANTHER database for alignment to specific cellular pathways. Fig. 2 is a pie chart indicating the pathways identified along with the percentage of the proteins corresponding to each fraction. The most prominent class of proteins were those involved in lipid, fatty acid and steroid metabolism (18%). Other functional groupings included metabolism (protein, carbohydrate and amino acid), electron transport, immunity and defence, and transport. In order to mine further into the specific pathways influenced by Nrf2, the 108 proteins identified as Nrf2-regulated from iTRAQ analysis 1 were subjected to pathway analysis using the software MetaCore. Of the 108 proteins, 104 were recognised by MetaCore and 68 had been mapped to pathways. The significant proteins were analysed against a background file containing all proteins quantified across the four replicate runs. Of the 769 proteins in the background file, 752 were recognised by the software and 504 had been mapped to pathways. Ten pathways were identified as significantly different (*p* < 0.05) between wild type and Nrf2 null mice, as shown in Table 5: seven of these are involved in fatty acid metabolism or other lipid-related processes. In particular, several of the pathways are associated with peroxisomes, suggesting that non-mitochondrial fatty acid metabolism may be a specific target for Nrf2-associated protein expression. Amongst the ten significant pathways identified only one, the glutathione metabolism pathway, is directly involved in cellular defence against reactive oxygen species or electrophiles. Nevertheless, this pathway was populated by five differentially regulated proteins (Table 5).

#### 3.1.4 ATP-citrate lyase is negatively regulated by Nrf2 in mice

**Fig. 3:**
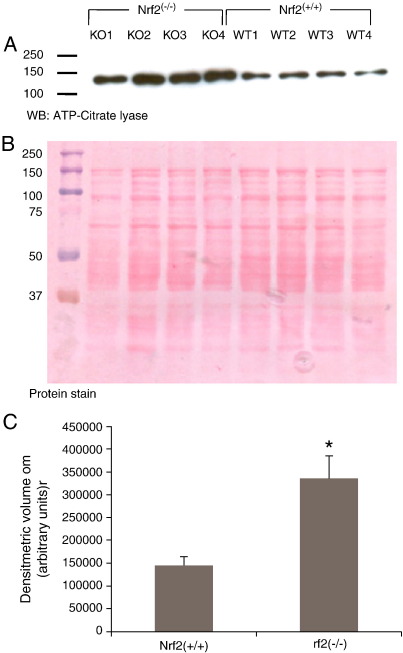
Western immunoblot of ATP-citrate lyase in liver homogenate from Nrf2^(−/−)^ and Nrf2^(+/+)^ mice. (A) Immunoblot for ATP-citrate lyase in liver homogenate from four Nrf2^(−/−)^ mice (KO1–KO4) and four Nrf2^(+/+)^ mice (WT1–WT4). The molecular mass of ATP-citrate lyase is approximately 120 kDa. (B) Ponceau protein stain of the transfer membrane shown in A) indicating approximately equal loading across the gel. Lane KO1 shows slightly decreased loading which is consistent with the lower level of ATP-citrate lyase in the blot above. (C) Densitometric analysis of immunoblot showing a statistically significant (*p* < 0.05; Student's *t*-test) elevation of expression in the Nrf2^(−/−)^ mice compared with the wild type controls.

Since a role for Nrf2 as a negative regulator of proteins involved in lipid metabolism has only recently been suggested [48,49], an attempt was made to verify some of the changes observed by immunoblotting. One of the most significant differences observed in the experiments in iTRAQ analysis 1 involved ATP-citrate lyase. This showed a mean 1.75-fold increase but in the “test” cohort a value of 1.2-fold was seen, which failed to reach significance (data not shown). Consequently, a comparison between the wild type and knockout animals was conducted by Western immunoblotting in order to validate the original iTRAQ observation. Due to the difficulty in identifying a suitable ‘housekeeping’ protein (we have found both actin and GAPDH to be unreliable when comparing whole liver homogenates from Nrf2^(^^−^^/^^−^^)^ and Nrf2^(+/+)^ mice) the total protein (Ponceau) stain was used as a loading control. The immunoblot (Fig. 3) confirmed that ATP-citrate lyase was indeed considerably over-expressed in Nrf2-null mouse liver with densitometric analysis indicating a 2.6-fold enhancement. As far as we are aware, this association between ATP-citrate lyase and Nrf2 has not been shown previously.

### 3.2 Identification of Nrf2-dependent liver proteins: 2-DE studies

**Fig. 4:**
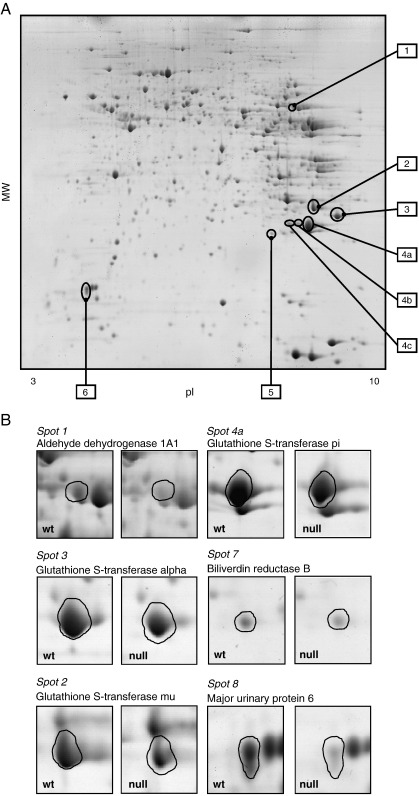
2D gel electropherograms of Nrf2 null and wild type mouse liver proteins. (A) Representative 2DE gel of liver homogenate prepared from a wild type mouse annotated with the spot reference numbers of proteins that were found to be regulated by Nrf2. (B) Expanded montages of differentially expressed protein spots from Nrf2^(+/+)^ and Nrf2^(−/−)^ mouse liver homogenates.

Mouse liver protein extracts were separated by 2-DE and the protein spots were visualized following staining with colloidal Coomassie blue. A total of 8 gels were produced (4 Nrf2^(+/+)^ , 4 Nrf2^(^^−^^/^^−^^)^). Approximately 500 spots/gel were detected by the automated spot detection algorithm across the 8 gels. Using the criteria defined above (statistically significant difference within one or more comparisons and spot detected in all four gels for any treatment group) 8 spots were differentially expressed indicating a role for Nrf2 in their regulation. These spots are labelled in the representative gel image (from a wild type control mouse liver) shown in Fig. 4a. Montage images of the differentially expressed spots across the four treatment groups are shown in Fig. 4b. Table 6 lists all the gel spots whose intensity varied in one or more of the treatment groups. Proteins associated with each gel spot were identified by MALDI mass spectrometric analysis. One protein (glutathione S-transferase pi) was identified in three of the differentially regulated spots.

**Fig. 5:**
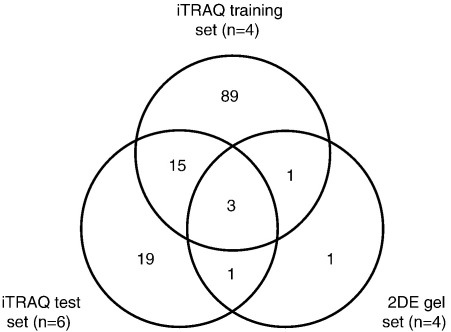
Venn diagram indicating the overlap between the proteins identified as Nrf2-regulated across the three different analyses.

Attempts to relate the iTRAQ data with the 2DE gel protein expression changes were hampered by the small number of proteins identified by the gel-based approach. This may reflect the fact that Nrf2-regulated proteins have properties that are not amenable to 2DE gel analysis, e.g. membrane bound, low abundance or incompatible pKa values. Only two of the identified proteins, glutathione S-transferases Mu1 and Pi1, were shown to be Nrf2-regulated in both the 2DE analysis and the two iTRAQ analyses (Table 7). A summary of the overlap between the three different analyses is provided by Venn diagram in Fig. 5.

### 3.3 Identification of putative antioxidant response elements (ARE) and ARE-related motifs in the promoters of the Nrf2-regulated genes

Each of the genes encoding proteins identified as Nrf2-regulated was interrogated for ARE or ARE-like enhancer elements in their promoter regions. Using a string-based searching algorithm with the input term RTGABNNNTCA (representing the consensus sequence derived by Nioi et al. [44]), several ARE sequences were identified across the panel of genes shown to be differentially expressed between Nrf2^(+/+)^ and Nrf2^(^^−^^/^^−^^)^ mice. Table 8 shows the number of consensus sequences identified in the 2000 bp promoter regions of the genes encoding nine representative proteins whose expression showed the greatest differential expression (> 0.4-fold difference) between the two mouse strains. It is apparent that there is little correlation between the number of perfect AREs identified and the fold-change in expression. Indeed, the average number of consensus ARE sequences identified by string-based searching across the entire panel of proteins identified was 1.21 compared with a value of 1.25 for those shown to be Nrf2 regulated. The complete data set for promoter analysis of all proteins significantly altered before correction for multiple testing is given in Table 1 of the on-line supplementary data.

For the matrix analysis, the *patser* algorithm assigns a score for each region within the promoter that matches the position-specific probability matrix. The score is based on the degree of similarity to the most frequently observed sequence within a series of known Nrf2 target genes [45]. To define a reference score, promoters for all the 769 quantified proteins were searched for putative ARE sequences. The mean *patser* score across the entire panel of genes was 2.50 whilst that for the Nrf2-regulated genes was 2.03.

## 4 Discussion

Nrf2 deficient mice are highly susceptible to liver damage evoked by a range of chemical hepatotoxins [25,30–34]. The cause of this predisposition may be either reduced constitutive expression of Nrf2-regulated genes or the loss of ability to respond to the noxious stimulus by up-regulation of cellular defence proteins. It is likely that both of these potential mechanisms plays a role in counteracting the damage caused by exposure to chemical toxins, however, as yet the relative importance of each pathway has not been established for individual hepatotoxins. Therapeutically, it is important to understand how each mechanism contributes to the cellular defence process since the Nrf2/Keap1 system provides a potential focus for the development of therapeutic strategies for management of drug or chemical-induced liver pathologies.

In most of the studies conducted with hepatotoxins in Nrf2 null mice, the chemical was administered either acutely or over a short dosing period. For example, loss of protection against liver damage from paracetamol can be observed in Nrf2 null mice following a single hepatotoxic dose. Although we have previously shown that paracetamol can activate hepatic Nrf2, even at doses that do not give rise to overt toxicity [50], it seems unlikely that such transcriptional activation, and the consequent up-regulation of cellular defence proteins, could occur sufficiently rapidly to afford protection against a massive acute chemical insult. Overt liver damage can be seen within 5 h following a toxic dose of paracetamol in mice: a timescale inconsistent with the up-regulation of proteins involved in the defence response [51]. Whilst the induction of Nrf2 may play a role in damage limitation and repair, it seems likely that constitutive differences between the wild type and knockout animals represent the major factor in protecting against the initial hepatotoxic response. Although several transcriptomic studies incorporate a comparison between Nrf2 wild type and null mice, none has specifically addressed the differences at the constitutive level. Consequently, this study represents the first comprehensive global analysis of the role of Nrf2 in the basal regulation of proteins in the liver.

The main aim of the study was to identify protein networks that are perturbed at the constitutive level in Nrf2 null mice compared with wild type controls. In addition, we attempted to produce a list of differentially expressed proteins that could be employed as definitive indices of Nrf2 activity and, thus, provide a pool of potential biomarkers for application in preclinical drug safety assessment. Such potential biomarkers might ultimately provide a translational bridge for assessment of Nrf2 activity in man. By using an experimental approach incorporating three independent sample cohorts we identified twenty proteins that were Nrf2-regulated in at least two of the three independent analyses. Of these, twelve proteins were down-regulated in Nrf2 null mice, seven of which are involved in drug metabolism and, predominantly, phase II metabolism. The reproducible and substantial reduction of proteins such as glutathione S-transferases mu and pi, and the UDP-glucuronosyl transferase 2B5 in Nrf2^(^^−^^/^^−^^)^ mice clearly indicates that protection against chemical toxins that undergo bioactivation to chemically reactive species, such as electrophiles, may be severely compromised due to lack of Nrf2 under basal conditions. The constitutive deficiency in such protective proteins will almost certainly play a part in the enhanced susceptibility to chemical toxins seen upon acute administration.

Somewhat surprisingly, many proteins that were significantly different between the null and wild-type animals were up-regulated in the absence of Nrf2, suggesting a negative regulation of their expression. Inspection of these proteins indicated that the majority were primarily involved in lipid metabolism. Consequently, an attempt was made to categorise the differentially expressed proteins with respect to biochemical function by pathway analysis. MetaCore. These analyses identified lipid metabolic pathways as being highly overrepresented within the lists of significantly altered proteins when compared against the entire list of proteins identified. Indeed, seven of the ten pathways shown to be significantly perturbed in the Nrf2^(^^−^^/^^−^^)^ mice related to the regulation of lipid biochemistry — in particular with respect to lipogenesis.

A potential role for Nrf2 in the regulation of lipid biochemistry, and more specifically in the disposition of fatty acids, has only recently been recognised [48,49,52]. This probably reflects the fact that earlier studies with transgenic animals predominantly concentrated on proteins that are directly correlated with Nrf2 activity, which comprises principally proteins involved in cellular antioxidant defence. Three recent studies have, however, noted the reciprocal relationship between Nrf2 function and the expression of multiple lipid-related gene products. Studies by Tanaka et al. [53], involving Nrf2^(^^−^^/^^−^^)^ mice fed a high fat diet, and Yates et al. [54], which compared Keap1 knockout mice with mice exposed to a potent Nrf2 inducer, utilized transcriptomic approaches to define gene expression profiles. Both studies noted that a high proportion of the up-regulated mRNAs coded for proteins involved in lipid homeostasis. In the former study, 4 weeks on a high fat diet resulted in a marked increase in the mRNAs for several cholesterol synthetic and up-take genes, including LDL receptor, HMGcoA reductase, HMGCoA synthase and SR-B1. Interestingly, the mRNA for Nrf2 itself was substantially reduced following this diet, suggesting that the Keap1/Nrf2 pathway may be directly regulated by certain dietary lipids. This must be balanced against the fact that some terpenoids, which are also lipids and share synthetic pathways with cholesterol, are among the most potent activators of Nrf2 in mouse models [55,56]. For example, the synthetic triterpenoid CDDO-Im has been shown to reduce hepatic lipid accumulation in mice on a high fat diet through activation of Keap1/Nrf2 signalling [49]. Clearly, the role of Nrf2 in lipid homeostasis is complex and requires further clarification. One of the most recent demonstrations that Nrf2 is negatively linked to the expression of lipid-related genes results from a study by Chowdary et al. [48] investigating the influence of Nrf2 on non-alcoholic hepatosteatosis (NASH). This disease is characterised by macro- and/or micro-vesicular vacuolization of hepatocytes and can be induced by administering a methionine/choline deficient diet. The histopathological symptoms of the condition were exacerbated in Nrf2 deficient mice and was accompanied by up-regulation of proteins involved in lipid metabolism including Adrp, a fatty acid- and cholesterol-binding protein that promotes accumulation of triacylglycerols and stimulates the uptake of fatty acids [57].

One of the proteins involved in lipid metabolism shown to be strongly enhanced in Nrf2^(^^−^^/^^−^^)^ mice in the current study was ATP citrate synthase (also known as ATP-citrate lyase). The almost two-fold increase in this enzyme indicated by the initial iTRAQ analysis (Table 2) was confirmed by Western immunoblotting (Fig. 3). As far as we are aware, regulation of this protein by Nrf2 has not been previously demonstrated. ATP-citrate lyase plays a critical role in acetyl CoA production within the cytoplasm of most cells, and is especially active in liver [58]. In the presence of ATP and Coenzyme A, ATP-citrate lyase is able to cleave citrate to form acetyl-CoA and oxaloacetic acid. The latter is a precursor for pyruvate which sits at the crossroads of multiple biochemical pathways, such as amino acid synthesis, glycogenolysis and lipogenesis. Furthermore, it has recently been shown that ATP-citrate lyase is a key enzyme in the acetylation of histones, and may therefore play a major role in gene transcription [59,60]. The demonstration that loss of Nrf2 results in such a large up-regulation of this already abundant protein may therefore have significant implication for multiple cellular functions, and this is the subject of further investigation in our laboratories.

Of all the statistically significant changes in protein expression between wild type and Nrf2 null mice, numerically the largest fold decrease was seen with major urinary protein 6 (MUP6; 0.35 relative to wild type) whilst the biggest increase was seen with epidermal fatty acid binding protein (FABP5; 2.97). MUP6 is a member of a species and sex specific class of secreted proteins synthesised in the liver but used by male mice for a variety of behavioural purposes, including territorial marking and mate attraction [61]. Major urinary proteins (MUPs) are lipid binding molecules that are specifically tailored to the transport of pheromones: following urinary excretion the pheromones are released only slowly from the MUP to prolong their signalling properties. Curiously, fatty acid binding proteins belong to the same class of proteins as the MUPs — both are lipocalins — and they fulfil a similar function, both being involved in lipid transport. Consequently, the two proteins whose expression was most disparately affected by loss of Nrf2 were of a similar type, again emphasising the complexity of Nrf2 regulation of lipid metabolism. It is possible that up regulation of FABP5 occurred at the expense of MUP6 synthesis, which then showed a marked fall in expression; further work is required to understand the interaction between these proteins and the precise role of Nrf2. Perturbation of the expression of FABPs and MUPs has been shown in other mouse models involving antioxidant proteins. CuZn superoxide dismutase (Sod1) deficient mice showed a marked decrease in MUP11 and MUP8 expression, but in this case FABP1 was also down-regulated [62].

A requisite property of all genes identified thus far to be Nrf2-responsive is that they contain an ARE sequence. Consequently, it was important to seek the presence of such AREs within the promoter regions of the genes encoding the identified proteins, particularly those not previously reported to be Nrf2-dependent. This was accomplished using software available in the public domain that allows both multiple string searching and pattern recognition analysis of 5′-flanking regions. String searching based on the ‘perfect’ ARE sequence of RTGABNNNGA as defined by Rushmore [63–66] indicated that a wide variety of potential AREs was present across the 16 genes significantly altered in Nrf2 null animals. However, only GSTM1 among the most highly Nrf2 regulated genes had more than two perfect ARE sequences within their promoter regions. Overall, there was little difference in the average number of AREs found in the Nrf2-regulated genes (mean = 1.25) compared with the number found right across the pool of identified proteins (1.21). Analysis based on a pattern recognition algorithm (*patser*) similarly showed little difference between up-or down-regulated genes when compared against the total protein pool. These results suggest that prediction of Nrf2-regulated genes based on regulatory sequence analysis may be an unreliable approach and a molecular-based promoter analysis is required to define the precise site of Nrf2 activation.

In summary, this study has identified a panel of Nrf2-dependent hepatic proteins that is statistically robust and demonstrated both decreased and enhanced expression in the absence of the Nrf2 gene. Twenty proteins were identified as Nrf2-regulated in at least two of the three analyses providing further confidence that these proteins might provide useful candidate biomarkers for future translational studies. The number of proteins that can be interrogated by currently available proteomic technology is far fewer than the number of genes interrogated in the oligoarray studies carried out by various groups [37–39]. This reflects, at least in part, the much lower analyte coverage of proteomic approaches and the fact that enhanced mRNA levels are often not mirrored by an equivalent up-regulation of the corresponding protein [41]. From a global perspective, there was little concordance between the proteins identified here and in all the various microarray studies presented previously. However, there was some overlap with well-established Nrf2-regulated mouse genes, such as glutathione transferases and glucuronyl transferases. Overall, the number of phase II proteins shown in the study to be Nrf2-regulated was surprisingly small and only one pathway (glutathione metabolism) involved in cellular defence was identified as Nrf2 regulated from the MetaCore analysis. iTRAQ suffers from the same limitation as all global proteomic methodologies in that, although it can sample around 1000 proteins simultaneously, high abundance species still dominate. Furthermore, the inherent variability associated with any technique that relies on the quantification of a protein based on the analysis of multiple peptide mass spectra (where the number of peptides identified may vary between analyses) means that fold changes less than 20% are unlikely to yield statistically robust quantitative data. In biological terms, however, a 10–20% decrease in protein expression could have a marked bearing on the toxicological consequence of chemical exposure. However, the strength of such global approaches is that they can reveal hitherto unrecognised roles for key cellular regulators, as evidenced here by the demonstration that lipid homeostasis is a key function of Nrf2. In conclusion, this study has identified a reliable panel of proteins that are reproducibly Nrf2-regulated at the constitutive level. This paves the way for the future translational applications in the pursuit of a human biomarker that would enable the assessment of the role of Nrf2 in man. The identification of two principal sets of seemingly unrelated proteins — one involved in cellular defence, the other in lipid homeostasis — that are reciprocally regulated by Nrf2 was an unexpected finding of this study, and may provide a powerful reservoir of diagnostic biomarkers for development in both animal and human studies of Nrf2 activity.

## Figures and Tables

**Table 1:** Total numbers of proteins identified and quantified with a false discovery rate (FDR) exclusion of 1% in iTRAQ analyses 1 and 2.

iTRAQ analysis	LC-MS analysis	No. of proteins identified	No. of proteins identified above 1% global FDR	No. of proteins quantified
1	Run 1	486	265	162
Run 2	1287	911	620
Run 3	1003	759	593
Run 4	726	563	426
Total		1654	1109	769

2	Run 1	1068	825	654
Run 2	1065	780	661
Run 3	1068	711	637
Total		1717	1070	628

Numbers are given for proteins identified with a confidence greater than 90% and for those characterized by at least 2 peptides. The number of proteins quantified relates to those proteins determined in all eight mouse liver samples.

**Table 2:** Nrf2-regulated mouse hepatic proteins identified in iTRAQ analysis 1.

					Relative expression compared to WT 1															
					Nrf2^(+/+)^							Nrf2^(−/−)^							Fold change	
SwissProt acc. no.	Name	*n*	Average no. of peptides	Average coverage (%)	Mouse WT1	MouseWT2	Mouse WT3	Mouse WT4	Geometric mean	Lower 95% CI	Upper 95% CI	Mouse KO1	Mouse KO2	Mouse KO3	Mouse KO4	Geometric mean	Lower 95% CI	Upper 95% CI	Nrf2^(−/−)^ Nrf2^(+/+)^	BH *p*
P02762	Major urinary protein 6	4	19.8	54.9	1.00	1.35	1.29	1.54	1.28	1.07	1.53	0.47	0.28	0.48	0.64	0.45	0.32	0.63	0.35	0.057
P17427	AP-2 complex subunit alpha2	1	1.0	2.5	1.00	1.25	1.93	1.51	1.38	1.05	1.82	0.46	0.43	0.71	0.66	0.55	0.43	0.71	0.40	0.064
P10649	Glutathione S-transferase Mu 1	4	13.8	39.2	1.00	1.31	1.00	1.11	1.10	0.97	1.24	0.47	0.53	0.44	0.42	0.46	0.42	0.51	0.42	0.009
Q61656	Probable ATP-dependent RNA helicase DDX5	1	2.0	5.4	1.00	1.31	1.19	1.35	1.20	1.05	1.38	0.37	0.85	0.51	0.79	0.59	0.40	0.87	0.49	0.148
Q91WG8	Bifunctional UDP-N-acetylglucosamine 2-epimerase	1	2.0	4.0	1.00	0.98	1.13	1.19	1.07	0.98	1.17	0.49	0.60	0.68	0.59	0.59	0.52	0.67	0.55	0.022
P19157	Glutathione S-transferase P 1	4	43.0	76.3	1.00	1.21	0.94	1.12	1.06	0.95	1.19	0.62	0.56	0.60	0.54	0.58	0.55	0.62	0.55	0.011
P17717	UDP-glucuronosyltransferase 2B5	4	5.8	15.5	1.00	1.16	0.99	1.08	1.05	0.98	1.13	0.59	0.57	0.56	0.61	0.58	0.56	0.61	0.55	0.004
Q63836	Selenium-binding protein 2	4	26.0	47.9	1.00	1.26	0.99	1.48	1.17	0.96	1.41	0.61	0.59	0.67	0.72	0.65	0.59	0.71	0.55	0.051
Q8VCC2	Liver carboxylesterase 1	3	2.3	4.6	1.00	1.34	1.06	0.94	1.08	0.93	1.25	0.62	0.60	0.58	0.70	0.62	0.58	0.68	0.58	0.042
Q60991	Cytochrome P450 7B1	1	2.0	7.1	1.00	1.43	1.66	1.65	1.40	1.11	1.77	0.85	0.82	0.82	0.79	0.82	0.80	0.84	0.58	0.073
P46425	Glutathione S-transferase P2	1	39.0	71.0	1.00	0.70	0.76	0.61	0.75	0.61	0.93	0.47	0.45	0.43	0.43	0.44	0.43	0.46	0.59	0.063
P24472	Glutathione S-transferase A4	2	2.5	17.6	1.00	1.01	0.99	0.92	0.98	0.94	1.02	0.49	0.62	0.76	0.50	0.58	0.48	0.72	0.60	0.073
O35660	Glutathione S-transferase M6	1	7.0	24.3	1.00	0.68	0.67	0.89	0.80	0.66	0.97	0.50	0.69	0.42	0.40	0.49	0.38	0.62	0.61	0.179
P00186	Cytochrome P450 1A2	3	3.0	10.9	1.00	1.14	1.26	1.21	1.15	1.04	1.27	0.59	0.61	0.91	0.86	0.73	0.58	0.91	0.63	0.186
Q9EQU5	Protein SET	1	1.0	6.2	1.00	1.22	1.34	0.99	1.13	0.97	1.31	1.05	0.63	0.57	0.71	0.72	0.56	0.94	0.64	0.199
Q91X77	Cytochrome P450 2C50	3	6.0	16.5	1.00	1.30	1.29	1.33	1.22	1.07	1.40	0.67	0.67	1.03	0.87	0.80	0.65	0.98	0.65	0.162
Q6XVG2	Cytochrome P450 2C54	4	3.5	8.5	1.00	1.00	0.96	1.04	1.00	0.97	1.03	0.54	0.70	0.77	0.77	0.69	0.58	0.81	0.69	0.090
Q91XE8	Transmembrane protein 205	2	1.5	11.4	1.00	0.67	0.70	0.60	0.73	0.58	0.91	0.49	0.47	0.49	0.57	0.50	0.46	0.55	0.69	0.153
P15105	Glutamine synthetase	4	9.8	25.1	1.00	1.16	1.06	1.29	1.12	1.01	1.25	0.70	0.67	0.99	0.83	0.79	0.66	0.93	0.70	0.182
O55060	Thiopurine S-methyltransferase	2	1.0	5.4	1.00	0.85	0.99	0.75	0.89	0.78	1.02	0.49	0.71	0.71	0.70	0.65	0.54	0.77	0.72	0.194
O35490	Betaine-homocysteine S-methyltransferase 1	4	18.3	45.2	1.00	0.81	1.11	1.12	1.00	0.87	1.16	0.76	0.67	0.78	0.80	0.75	0.70	0.81	0.75	0.148
P24549	Retinal dehydrogenase 1	4	13.8	31.2	1.00	1.07	1.10	1.22	1.10	1.01	1.19	0.80	0.76	0.84	0.92	0.83	0.77	0.90	0.76	0.127
P06801	NADP-dependent malic enzyme	3	8.0	20.5	1.00	1.32	1.16	1.22	1.17	1.04	1.31	0.75	0.93	1.06	0.84	0.89	0.77	1.02	0.76	0.201
P62858	40 S ribosomal protein S28	4	1.0	17.4	1.00	1.03	1.08	1.11	1.05	1.01	1.10	0.87	0.76	0.81	0.82	0.82	0.77	0.86	0.77	0.038
Q91VA0	Acyl-coenzyme A synthetase ACSM1, mitochondrial	3	6.3	20.4	1.00	0.95	1.03	0.90	0.97	0.91	1.03	0.80	0.71	0.75	0.75	0.75	0.72	0.79	0.78	0.039
Q9JIF7	Coatomer subunit beta	2	3.0	3.9	1.00	0.86	0.92	0.94	0.93	0.87	0.99	0.77	0.74	0.65	0.74	0.72	0.67	0.77	0.78	0.044
O55125	Protein NipSnap homolog 1	3	1.0	4.0	1.00	0.76	0.80	0.93	0.87	0.77	0.98	0.62	0.68	0.68	0.73	0.68	0.64	0.72	0.78	0.201
Q99JI4	26 S proteasome non-ATPase regulatory subunit 6	2	1.0	3.9	1.00	0.76	0.75	0.74	0.81	0.70	0.93	0.65	0.67	0.60	0.61	0.63	0.60	0.67	0.78	0.182
Q99J99	3-mercaptopyruvate sulfurtransferase	2	2.0	10.9	1.00	0.99	0.87	0.94	0.95	0.89	1.01	0.72	0.71	0.80	0.79	0.75	0.71	0.80	0.79	0.057
Q76MZ3	Serine/threonine-protein phosphatase 2A 65 kDa regulatory subunit A alpha	2	1.0	3.4	1.00	0.76	0.94	0.83	0.88	0.78	0.99	0.65	0.73	0.60	0.82	0.70	0.61	0.80	0.80	0.204
Q9Z0X1	Apoptosis-inducing factor 1, mitochondrial	2	1.0	2.1	1.00	0.99	0.89	0.86	0.93	0.87	1.00	0.67	0.78	0.76	0.80	0.75	0.69	0.81	0.80	0.114
O70475	UDP-glucose 6-dehydrogenase	3	5.7	19.5	1.00	0.94	0.96	1.06	0.99	0.94	1.04	0.77	0.87	0.81	0.75	0.80	0.75	0.85	0.81	0.061
Q8R1G2	Carboxymethylene-butenolidase homolog	2	2.5	12.9	1.00	0.85	0.96	1.06	0.96	0.88	1.06	0.71	0.75	0.82	0.86	0.78	0.72	0.85	0.81	0.178
Q8VCU1	Liver carboxylesterase 31-like	3	10.0	20.4	1.00	0.95	0.90	0.83	0.92	0.85	0.99	0.74	0.69	0.79	0.78	0.75	0.70	0.79	0.81	0.117
Q8VCA8	Secernin-2	1	1.0	4.0	1.00	1.09	1.05	0.92	1.01	0.94	1.09	0.87	0.76	0.93	0.78	0.83	0.76	0.91	0.82	0.156
Q91VS7	Microsomal glutathione S-transferase 1	4	5.0	30.2	1.00	0.92	0.95	0.95	0.95	0.92	0.99	0.84	0.71	0.73	0.85	0.78	0.71	0.86	0.82	0.162
Q9D6Y7	Peptide methionine sulfoxide reductase	3	3.0	16.7	1.00	1.08	1.04	1.06	1.04	1.01	1.08	0.83	0.82	0.93	0.85	0.86	0.81	0.91	0.82	0.044
P70441	Na(+)/H(+) exchange regulatory cofactor NHE-RF1	3	1.7	5.7	1.00	0.82	0.84	0.73	0.84	0.74	0.96	0.75	0.68	0.69	0.68	0.70	0.67	0.73	0.83	0.198
Q8VCW8	Acyl-CoA synthetase family member 2, mitochondrial	3	6.3	18.5	1.00	0.98	1.00	0.93	0.98	0.94	1.01	0.81	0.77	0.85	0.82	0.81	0.78	0.85	0.83	0.030
P57776	Elongation factor 1-delta	3	3.7	23.5	1.00	0.90	0.87	0.83	0.90	0.83	0.97	0.84	0.76	0.78	0.74	0.78	0.74	0.82	0.87	0.180
P07759	Serine protease inhibitor A3K	3	9.0	24.5	1.00	1.03	1.16	1.05	1.06	1.00	1.13	0.91	0.91	0.87	0.97	0.92	0.88	0.96	0.87	0.123
Q91ZJ5	UTP-glucose-1-phosphate uridylyltransferase	3	3.0	8.0	1.00	0.99	1.08	1.06	1.03	0.99	1.08	0.86	0.89	0.96	0.88	0.90	0.85	0.94	0.87	0.156
P11352	Glutathione peroxidase 1	4	4.5	26.1	1.00	0.96	1.07	1.12	1.04	0.97	1.11	0.90	0.88	0.95	0.94	0.92	0.89	0.95	0.89	0.193
P60867	40 S ribosomal protein S20	3	2.3	16.2	1.00	0.91	1.01	0.91	0.96	0.90	1.01	0.90	0.81	0.86	0.84	0.85	0.82	0.89	0.89	0.178
Q9JII6	Alcohol dehydrogenase [NADP+]	4	5.5	25.0	1.00	0.95	0.97	0.89	0.95	0.91	1.00	0.85	0.89	0.86	0.84	0.86	0.84	0.88	0.90	0.121
Q9DBJ1	Phosphoglycerate mutase 1	2	8.0	44.3	1.00	0.98	1.02	1.04	1.01	0.98	1.03	1.09	1.06	1.04	1.10	1.07	1.05	1.10	1.07	0.128
Q8BVI4	Dihydropteridine reductase	3	3.0	18.4	1.00	1.09	1.05	1.09	1.06	1.02	1.10	1.14	1.12	1.15	1.16	1.14	1.12	1.16	1.08	0.178
Q8BH00	Aldehyde dehydrogenase family 8 member A1	3	13.3	31.9	1.00	1.07	1.08	1.14	1.07	1.02	1.13	1.19	1.19	1.14	1.15	1.17	1.14	1.20	1.09	0.206
Q8BFR5	Elongation factor Tu, mitochondrial	3	2.7	10.4	1.00	0.93	0.97	0.91	0.95	0.91	0.99	1.05	0.99	1.07	1.05	1.04	1.00	1.08	1.09	0.144
Q3UQ44	Ras GTPase-activating-like protein IQGAP2	3	3.3	3.4	1.00	1.04	0.99	0.96	1.00	0.97	1.03	1.11	1.15	1.09	1.08	1.10	1.08	1.13	1.11	0.057
P21107	Tropomyosin alpha-3 chain	1	1.0	3.5	1.00	1.00	1.03	1.05	1.02	1.00	1.04	1.12	1.23	1.04	1.16	1.14	1.06	1.22	1.12	0.188
Q64374	Regucalcin	4	13.8	42.6	1.00	1.07	1.02	1.08	1.04	1.01	1.08	1.12	1.22	1.24	1.09	1.17	1.10	1.24	1.12	0.148
P45952	Medium-chain specific acyl-CoA dehydrogenase, mitochondrial	3	4.7	14.2	1.00	1.06	1.11	1.08	1.06	1.02	1.11	1.20	1.20	1.24	1.13	1.19	1.15	1.23	1.12	0.095
P62991	Ubiquitin	4	4.8	50.3	1.00	1.08	1.03	1.15	1.06	1.00	1.13	1.17	1.27	1.18	1.15	1.19	1.14	1.24	1.12	0.178
Q8CHT0	Delta-1-pyrroline-5-carboxylate dehydrogenase, mitochondrial	3	7.0	18.0	1.00	1.05	1.09	0.97	1.03	0.98	1.08	1.17	1.20	1.23	1.05	1.16	1.09	1.24	1.13	0.193
Q99J08	SEC14-like protein 2	3	6.3	25.5	1.00	1.08	1.12	1.21	1.10	1.02	1.19	1.27	1.18	1.32	1.23	1.25	1.19	1.31	1.14	0.186
Q02053	Ubiquitin-like modifier-activating enzyme 1	4	3.5	5.6	1.00	1.05	1.00	1.04	1.02	1.00	1.05	1.10	1.19	1.24	1.14	1.16	1.11	1.22	1.14	0.073
O88569	Heterogeneous nuclear ribonucleoproteins A2/B1	4	4.5	12.5	1.00	0.98	1.03	1.08	1.02	0.98	1.07	1.22	1.17	1.10	1.16	1.16	1.12	1.21	1.14	0.090
Q9QXD6	Fructose-1,6-bisphosphatase	4	16.0	46.5	1.00	0.93	0.98	0.92	0.96	0.92	0.99	1.16	1.14	1.05	1.01	1.09	1.02	1.16	1.14	0.144
Q99JI6	Ras-related protein Rap-1b	2	1.0	6.5	1.00	1.02	0.99	1.01	1.00	1.00	1.01	1.21	1.20	1.08	1.10	1.14	1.08	1.21	1.14	0.121
P50580	Proliferation-associated protein 2G4	2	2.0	6.6	1.00	1.01	1.07	0.98	1.01	0.97	1.05	1.10	1.19	1.25	1.10	1.16	1.09	1.23	1.14	0.127
Q9R0Q7	Prostaglandin E synthase 3	2	1.5	13.1	1.00	1.05	1.11	1.10	1.06	1.01	1.11	1.17	1.14	1.21	1.36	1.21	1.12	1.31	1.14	0.199
Q9DCN2	NADH-cytochrome b5 reductase 3	3	6.0	27.9	1.00	1.00	0.98	0.98	0.99	0.98	1.00	1.18	1.05	1.11	1.21	1.13	1.07	1.21	1.15	0.072
Q99LP6	GrpE protein homolog 1, mitochondrial	2	1.0	6.5	1.00	1.18	1.10	1.09	1.09	1.02	1.17	1.31	1.22	1.20	1.31	1.26	1.20	1.32	1.15	0.142
Q9JI75	Ribosyldihydronicotinamide dehydrogenase [quinone]	2	3.0	19.3	1.00	0.88	0.98	0.91	0.94	0.88	1.00	1.16	1.12	1.02	1.05	1.08	1.02	1.15	1.15	0.144
P00329	Alcohol dehydrogenase 1	4	13.0	32.8	1.00	1.04	1.10	1.05	1.05	1.01	1.09	1.26	1.20	1.18	1.20	1.21	1.18	1.24	1.16	0.039
P06151	L-lactate dehydrogenase A chain	4	12.5	36.1	1.00	0.92	0.96	0.90	0.94	0.90	0.99	1.22	1.18	1.00	1.06	1.11	1.01	1.22	1.18	0.178
Q8CHR6	Dihydropyrimidine dehydrogenase [NADP+]	2	2.0	2.6	1.00	0.96	1.00	1.01	0.99	0.97	1.01	1.33	1.13	1.16	1.14	1.18	1.10	1.28	1.20	0.072
P00405	Cytochrome *c* oxidase subunit 2	2	2.5	15.2	1.00	1.16	0.98	0.99	1.03	0.95	1.11	1.19	1.26	1.18	1.28	1.23	1.18	1.28	1.20	0.105
Q9QXE0	2-hydroxyacyl-CoA lyase 1	3	2.7	7.5	1.00	1.02	0.89	0.83	0.93	0.84	1.02	1.10	1.17	1.13	1.07	1.12	1.08	1.16	1.20	0.117
Q60932	Voltage-dependent anion-selective channel protein 1	2	1.5	6.6	1.00	1.01	0.95	1.05	1.00	0.96	1.04	1.24	1.22	1.22	1.16	1.21	1.18	1.25	1.21	0.022
Q61207	Sulfated glycoprotein 1	3	2.0	2.8	1.00	1.08	1.17	1.24	1.12	1.02	1.23	1.35	1.50	1.32	1.30	1.37	1.28	1.46	1.22	0.121
Q8VC12	Probable urocanate hydratase	4	8.0	14.9	1.00	1.20	1.12	1.09	1.10	1.02	1.18	1.36	1.39	1.30	1.33	1.35	1.31	1.39	1.23	0.063
P80316	T-complex protein 1 subunit epsilon	1	4.0	14.4	1.00	1.22	1.02	1.03	1.06	0.97	1.16	1.37	1.30	1.23	1.34	1.31	1.25	1.37	1.23	0.103
P50172	Corticosteroid 11-beta-dehydrogenase isozyme 1	4	2.8	10.2	1.00	1.19	0.98	1.09	1.06	0.98	1.16	1.31	1.20	1.41	1.32	1.31	1.23	1.39	1.23	0.103
Q8VCR7	Abhydrolase domain-containing protein 14B	3	3.7	22.1	1.00	1.17	1.01	1.24	1.10	0.99	1.22	1.36	1.36	1.31	1.41	1.36	1.32	1.40	1.24	0.123
Q9DD20	Methyltransferase-like protein 7B	3	3.0	15.2	1.00	0.93	0.90	1.05	0.97	0.90	1.04	1.19	1.23	1.16	1.21	1.20	1.17	1.23	1.24	0.044
Q61171	Peroxiredoxin-2	3	1.7	10.4	1.00	1.11	0.94	1.21	1.06	0.95	1.18	1.26	1.29	1.38	1.35	1.32	1.27	1.37	1.25	0.142
P24270	Catalase	4	12.3	25.9	1.00	1.25	1.02	1.14	1.10	0.99	1.21	1.39	1.33	1.41	1.40	1.38	1.35	1.42	1.26	0.096
P16460	Argininosuccinate synthase	4	26.8	47.6	1.00	0.82	1.02	0.89	0.93	0.84	1.03	1.26	1.31	1.03	1.11	1.17	1.05	1.31	1.26	0.162
P31786	Acyl-CoA-binding protein	4	4.8	39.1	1.00	0.85	0.92	0.83	0.90	0.83	0.97	1.22	1.20	1.05	1.09	1.14	1.06	1.22	1.26	0.073
Q61425	Hydroxyacyl-coenzyme A dehydrogenase, mitochondrial	3	3.3	12.1	1.00	1.04	0.98	1.04	1.01	0.99	1.04	1.09	1.25	1.51	1.31	1.28	1.12	1.46	1.26	0.156
A3KMP2	Tetratricopeptide repeat protein 38	3	2.3	6.4	1.00	0.95	0.96	1.16	1.02	0.93	1.11	1.21	1.41	1.24	1.33	1.29	1.21	1.39	1.27	0.117
Q99PG0	Arylacetamide deacetylase	3	3.3	12.8	1.00	1.13	1.13	1.12	1.09	1.03	1.16	1.44	1.25	1.36	1.54	1.39	1.28	1.52	1.27	0.072
P12787	Cytochrome *c* oxidase subunit 5A, mitochondrial	2	3.0	36.6	1.00	1.12	0.85	1.12	1.02	0.89	1.16	1.18	1.27	1.45	1.33	1.31	1.20	1.42	1.29	0.148
P32020	Non-specific lipid-transfer protein	4	11.8	25.1	1.00	1.34	1.09	1.22	1.15	1.02	1.31	1.53	1.41	1.45	1.54	1.48	1.42	1.55	1.29	0.117
P55096	ATP-binding cassette sub-family D member 3	3	2.3	5.8	1.00	1.29	1.08	1.29	1.16	1.02	1.32	1.46	1.37	1.53	1.64	1.50	1.39	1.61	1.29	0.142
P05201	Aspartate aminotransferase cytoplasmic	3	5.7	19.4	1.00	0.83	1.02	0.90	0.94	0.85	1.03	1.34	1.39	1.04	1.12	1.22	1.06	1.39	1.30	0.178
P19096	Fatty acid synthase	4	30.3	17.7	1.00	1.10	1.03	1.15	1.07	1.00	1.13	1.35	1.40	1.44	1.36	1.39	1.35	1.43	1.30	0.022
Q9R0H0	Peroxisomal acyl-coenzyme A oxidase 1	3	12.0	24.2	1.00	1.06	1.01	0.93	1.00	0.95	1.05	1.31	1.33	1.29	1.31	1.31	1.29	1.33	1.31	0.009
P17665	Cytochrome *c* oxidase subunit 7C, mitochondrial	1	2.0	47.6	1.00	0.89	0.87	1.07	0.95	0.87	1.05	1.34	1.15	1.34	1.26	1.27	1.18	1.36	1.33	0.072
Q9QXF8	Glycine N-methyltransferase	4	18.0	47.6	1.00	1.27	1.23	1.42	1.22	1.06	1.41	1.63	1.66	1.60	1.61	1.63	1.60	1.66	1.34	0.117
P35492	Histidine ammonia-lyase	3	6.7	13.1	1.00	1.08	1.17	1.12	1.09	1.02	1.17	1.57	1.54	1.33	1.44	1.47	1.36	1.58	1.34	0.044
P83940	Transcription elongation factor B polypeptide 1	1	1.0	8.0	1.00	1.02	0.87	1.11	1.00	0.90	1.10	1.40	1.31	1.33	1.31	1.34	1.30	1.38	1.35	0.050
P18242	Cathepsin D	2	5.0	17.7	1.00	1.20	1.00	1.42	1.14	0.97	1.35	1.49	1.86	1.47	1.48	1.57	1.40	1.75	1.37	0.178
P25688	Uricase	4	7.5	27.4	1.00	1.11	0.98	1.04	1.03	0.98	1.09	1.39	1.42	1.43	1.50	1.43	1.39	1.48	1.39	0.009
Q9QXD1	Peroxisomal acyl-coenzyme A oxidase 2	1	2.0	3.8	1.00	1.38	1.28	1.32	1.23	1.07	1.42	1.68	1.73	1.57	2.01	1.74	1.57	1.93	1.41	0.117
P62984	60 S ribosomal protein L40	1	1.0	19.2	1.00	0.96	0.93	1.07	0.99	0.93	1.05	1.42	1.52	1.41	1.27	1.40	1.31	1.51	1.42	0.022
Q99P30	Peroxisomal coenzyme A diphosphatase NUDT7	4	4.5	30.3	1.00	1.14	0.90	1.14	1.04	0.93	1.16	1.60	1.45	1.40	1.48	1.48	1.40	1.57	1.43	0.044
Q9DBM2	Peroxisomal bifunctional enzyme	4	2.3	4.7	1.00	1.35	1.10	1.20	1.16	1.02	1.31	1.52	1.76	1.60	1.73	1.65	1.54	1.76	1.43	0.057
O35423	Serine-pyruvate aminotransferase, mitochondrial	3	1.0	3.1	1.00	0.88	0.86	0.93	0.92	0.86	0.98	1.55	1.67	1.15	1.25	1.39	1.17	1.65	1.51	0.066
Q8VBT2	L-serine dehydratase	3	4.3	22.3	1.00	0.72	0.97	0.90	0.89	0.77	1.03	1.47	1.58	1.18	1.20	1.35	1.17	1.55	1.51	0.078
Q8JZR0	Long-chain-fatty-acid-CoA ligase 5	2	3.5	7.7	1.00	0.97	0.99	0.88	0.96	0.91	1.02	1.58	1.56	1.20	1.59	1.47	1.29	1.69	1.53	0.044
Q91V92	ATP-citrate synthase	3	11.3	14.4	1.00	1.13	1.05	1.09	1.07	1.01	1.12	1.97	2.02	1.84	1.79	1.90	1.80	2.01	1.78	0.003
P62827	GTP-binding nuclear protein Ran	1	1.0	8.8	1.00	1.69	1.44	1.61	1.41	1.12	1.78	2.37	2.79	3.07	2.11	2.56	2.17	3.01	1.82	0.101
P13516	Acyl-CoA desaturase 1	1	2.0	9.0	1.00	1.43	1.09	1.19	1.17	1.00	1.36	4.04	2.12	1.53	2.58	2.41	1.62	3.59	2.07	0.153
Q8VCH0	3-ketoacyl-CoA thiolase B, peroxisomal	3	6.7	25.2	1.00	1.89	1.43	1.44	1.41	1.09	1.82	2.92	3.61	4.04	2.98	3.35	2.88	3.91	2.39	0.044
Q05816	Fatty acid-binding protein, epidermal	4	1.8	17.0	1.00	1.24	1.01	0.85	1.02	0.87	1.18	3.64	3.17	2.74	2.62	3.02	2.61	3.50	2.97	0.009

Relative expression of hepatic proteins in livers of Nrf2 wild type (Nrf2^(+/+)^) and null (Nrf2^(−/−)^) mice determined in iTRAQ analysis 1. All values are expressed relative to a wild type control mouse (WT1). Proteins listed were significantly different in the null mice compared with wild type according to Student's *t*-test followed by Benjamini-Hochberg (BH) correction for multiple testing at a significance level of *p* ≤ 0.2. Four replicate iTRAQ analyses were conducted on each sample and the number of runs in which each protein appeared is designated by *n* in column 3. The values for each mouse thus represent the average of n replicates. The fold change was calculated from the geometric mean values obtained from the 4 individual mice. Variance of the geometric mean for the four animals in each group is expressed as upper and lower 95% confidence intervals (CI). Proteins are listed according to their expression in Nrf2^(−/−)^ mice relative to wild type animals in ascending order of the fold-change value.

**Table 3:** Differentially up-regulated proteins listed in the UniProt database as involved in lipid synthesis or metabolism in ITRAQ analysis 1.

SwissProt acc. no.	Name	Subcellular location	Relative expression compared to Nrf2^(+/+)^ mouse 1					
Nrf2^(+/+)^		Nrf2^(−/−)^		Fold change Nrf2^(−/−)^ Nrf2^(+/+)^	*p*
Geometric mean	95% CI	Geometric mean	95% CI
Q05816	Fatty acid-binding protein, epidermal	C	1.02	(0.87–1.18)	3.02	(2.61–3.50)	2.97	0.009
Q8VCH0	3-Ketoacyl-CoA thiolase B, peroxisomal	P	1.41	(1.09–1.82)	3.35	(2.88–3.91)	2.39	0.044
P13516	Acyl-CoA desaturase 1	ER	1.17	(1.00–1.36)	2.41	(1.62–3.59)	2.07	0.153
Q91V92	ATP-citrate synthase	C	1.07	(1.01–1.12)	1.90	(1.80–2.01)	1.78	0.003
Q8JZR0	Long-chain-fatty-acid-CoA ligase 5	ER, Mi	0.96	(0.91–1.02)	1.47	(1.29–1.69)	1.53	0.044
Q9DBM2	Peroxisomal bifunctional enzyme	P	1.16	(1.02–1.31)	1.65	(1.54–1.76)	1.43	0.057
Q99P30	Peroxisomal coenzyme A diphosphatase NUDT7	P	1.04	(0.93–1.16)	1.48	(1.40–1.57)	1.43	0.044
Q9QXD1	Peroxisomal acyl-coenzyme A oxidase 2	P	1.23	(1.07–1.42)	1.74	(1.57–1.93)	1.41	0.117
Q9R0H0	Peroxisomal acyl-coenzyme A oxidase 1	P	1.00	(0.95–1.05)	1.31	(1.29–1.33)	1.31	0.009
P19096	Fatty acid synthase	C	1.07	(1.00–1.13)	1.39	(1.35–1.43)	1.30	0.022
P32020	Non-specific lipid-transfer protein	C	1.15	(1.02–1.31)	1.48	(1.42–1.55)	1.29	0.117
Q61425	Hydroxyacyl-coenzyme A dehydrogenase, mitochondrial	Mi	1.01	(0.99–1.04)	1.28	(1.12–1.46)	1.26	0.156
P31786	Acyl-CoA-binding protein	Mi	0.90	(0.83–0.97)	1.14	(1.06–1.22)	1.26	0.073
P50172	Corticosteroid 11-beta-dehydrogenase isozyme 1	ER	1.06	(0.98–1.16)	1.31	(1.23–1.39)	1.23	0.103
Q9QXE0	2-Hydroxyacyl-CoA lyase 1	P	0.93	(0.84–1.02)	1.12	(1.08–1.16)	1.20	0.117

**Table 4:** Nrf2-regulated mouse hepatic proteins determined in iTRAQ analysis 2 (test set).

SwissProt acc. no.	Name	Relative expression compared to Nrf2^(+/+)^ mouse 1						Fold change Nrf2^(−/−)^ /Nrf2^(+/+)^	*p*
Nrf2^(+/+)^			Nrf2^(−/−)^		
Geometric mean	Lower 95% CI	Upper 95% CI	Geometric mean	Lower 95% CI	Upper 95% CI
P10649	Glutathione S-transferase Mu 1	1.00	0.91	1.10	0.44	0.40	0.47	0.44	0.001
P17717	UDP-glucuronosyltransferase 2B5	0.99	0.93	1.06	0.55	0.52	0.57	0.55	0.001
Q8VCC2	Liver carboxylesterase 1	1.06	0.96	1.17	0.59	0.54	0.64	0.56	0.001
Q91X77	Cytochrome P450 2C50	0.97	0.87	1.08	0.56	0.46	0.69	0.58	0.001
P19157	Glutathione S-transferase P 1	0.95	0.89	1.01	0.58	0.54	0.63	0.62	0.001
Q9D379	Epoxide hydrolase 1	0.97	0.90	1.05	0.63	0.59	0.68	0.65	0.001
Q64458	Cytochrome P450 2C29	1.08	0.90	1.29	0.75	0.62	0.90	0.69	0.001
P30115	Glutathione S-transferase A3	1.03	0.98	1.08	0.72	0.66	0.78	0.70	0.001
P24549	Retinal dehydrogenase 1	0.94	0.86	1.04	0.68	0.58	0.80	0.72	0.021
O70475	UDP-glucose 6-dehydrogenase	1.09	0.97	1.23	0.79	0.63	0.99	0.73	0.183
Q62452	UDP-glucuronosyltransferase 1-9	0.99	0.93	1.06	0.73	0.58	0.92	0.74	0.183
Q91VA0	Acyl-coenzyme A synthetase ACSM1, mitochondrial	0.97	0.91	1.04	0.79	0.74	0.84	0.81	0.001
Q64442	Sorbitol dehydrogenase	1.02	0.92	1.13	0.84	0.78	0.91	0.83	0.081
P97494	Glutamate-cysteine ligase catalytic subunit	1.15	1.06	1.25	0.95	0.89	1.02	0.83	0.021
Q8CG76	Aflatoxin B1 aldehyde reductase member 2	1.06	1.01	1.11	0.88	0.81	0.96	0.83	0.013
Q9CQX2	Cytochrome b5 type B	1.01	0.91	1.13	0.85	0.76	0.94	0.83	0.197
Q9JII6	Alcohol dehydrogenase [NADP+]	1.01	0.98	1.04	0.86	0.80	0.92	0.85	0.003
Q8VCW8	Acyl-CoA synthetase family member 2, mitochondrial	1.00	0.93	1.08	0.86	0.79	0.93	0.86	0.132
O55022	Membrane-associated progesterone receptor component 1	1.03	0.96	1.11	0.89	0.81	0.98	0.86	0.207
P47738	Aldehyde dehydrogenase, mitochondrial	1.02	0.99	1.06	0.88	0.85	0.92	0.86	0.000
Q8QZS1	3-hydroxyisobutyryl-CoA hydrolase, mitochondrial	1.07	1.00	1.13	0.94	0.89	1.00	0.89	0.084
Q9ET01	Glycogen phosphorylase, liver form	0.98	0.96	1.00	0.87	0.80	0.94	0.89	0.081
O35945	Aldehyde dehydrogenase, cytosolic 1	0.97	0.93	1.01	0.86	0.83	0.89	0.89	0.024
Q8VDJ3	Vigilin	1.10	1.05	1.15	0.99	0.94	1.04	0.90	0.069
Q9EQ20	Methylmalonate-semialdehyde dehydrogenase [acylating], mitochondrial	1.00	0.98	1.03	0.92	0.87	0.98	0.92	0.140
Q9Z2I8	Succinyl-CoA ligase [GDP-forming] subunit beta, mitochondrial	1.02	0.99	1.06	0.96	0.92	0.99	0.93	0.121
Q99P30	Peroxisomal coenzyme A diphosphatase NUDT7	0.98	0.93	1.03	1.10	1.05	1.17	1.13	0.039
Q9CW42	MOSC domain-containing protein 1, mitochondrial	0.95	0.90	1.00	1.09	1.04	1.15	1.15	0.095
Q9QXD6	Fructose-1,6-bisphosphatase 1	1.02	0.96	1.09	1.21	1.10	1.33	1.18	0.117
P31786	Acyl-CoA-binding protein	1.01	0.94	1.07	1.19	1.06	1.34	1.18	0.207
P24369	Peptidyl-prolyl cis-trans isomerase B	0.95	0.89	1.01	1.12	1.05	1.20	1.18	0.017
Q8VDM4	26 S proteasome non-ATPase regulatory subunit 2	0.90	0.80	1.02	1.08	1.00	1.17	1.20	0.183
P06151	L-lactate dehydrogenase A chain	0.97	0.89	1.05	1.16	1.06	1.28	1.20	0.086
Q61207	Sulfated glycoprotein 1	0.94	0.84	1.05	1.14	1.07	1.21	1.21	0.057
P16460	Argininosuccinate synthase	1.02	0.93	1.13	1.27	1.16	1.40	1.25	0.038
Q3THE2	Myosin regulatory light chain MRLC2	1.13	1.03	1.24	1.46	1.25	1.70	1.29	0.117
Q8VBT2	L-serine dehydratase	1.02	0.91	1.15	1.37	1.13	1.67	1.34	0.183
Q05816	Fatty acid-binding protein, epidermal	1.17	0.96	1.43	2.10	1.69	2.60	1.79	0.005

All values are expressed relative to a wild type control mouse (WT1). Proteins listed were significantly different in the null mice compared with wild type controls (Benjamini–Hochberg; *p* ≤ 0.2).

**Table 5:** Metacore network analysis of data from iTRAQ analysis 1.

	Pathway name	Negative log *p* value	Number of pathway objects
1	n-6 Polyunsaturated fatty acid biosynthesis	2.52	5
2	n-3 Polyunsaturated fatty acid biosynthesis	2.52	5
3	Regulation of lipid metabolism_Regulation of lipid metabolism via LXR, NF-Y and SREBP	2.44	3
4	Vitamin E (alfa-tocopherol) metabolism	1.98	5
5	Regulation of metabolism_Bile acids regulation of glucose and lipid metabolism via FXR	1.89	4
6	Fatty Acid Omega Oxidation	1.64	4
7	Peroxysomal straight-chain fatty acid beta-oxidation	1.64	4
8	CFTR-dependent regulation of ion channels in Airway Epithelium (norm and CF)	1.62	2
9	Cell cycle_Role of SCF complex in cell cycle regulation	1.62	2
10	Glutathione metabolism/Rodent version	1.3	5

Proteins identified in iTRAQ analysis 1 as being differentially expressed (Benjamini-Hochberg *p* ≤ 0.2) were interrogated for pathway perturbation using the pathway analysis software Metacore. The total list of all quantified proteins was applied as a background for the analysis.

**Table 6:** Proteins regulated by Nrf2 identified by 2DE analysis.

Protein spot	SwissProt acc. No.	Protein	Mr/pI	Normalized spot Intensity (% total spot intensity)		Fold change	*p*
				Nrf2^(+/+)^	Nrf2^(−/−)^	(Nrf2^(−/−)^/ Nrf2^(+/+)^)	
1	P24549	Aldehyde dehydrogenase family 1, subfamily A1	54468/7.91	0.19 ± 0.01	0.15 ± 0.01	0.78	0.0025
2	P10649	Glutathione S-transferase, mu 1	25970/7.7	0.88 ± 0.20	0.53 ± 0.07	0.60	0.0158
3	P30115	Glutathione S-transferase, alpha 3	25361/8.76	0.85 ± 0.09	0.67 ± 0.02	0.80	0.0098
4a	P19157	Glutathione S-transferase, pi 1	23609/7.69	2.06 ± 0.17	1.20 ± .50	0.58	0.0170
4b	P19157	Glutathione S-transferase, pi 1	23609/7.69	0.29 ± 0.03	0.017 ± 0.05	0.57	0.0040
4c	P19157	Glutathione S-transferase, pi 1	23609/7.69	0.21 ± 0.01	0.11 ± 0.05	0.50	0.0073
5	Q923D2	Biliverdin reductase B	22197/6.49	0.13 ± 0.01	0.010 ± 0.01	0.83	0.0155
6	P11588	Major urinary protein 6	20648/5.0	0.46 ± 0.06	0.17 ± 0.05	0.36	0.0002

Proteins from the livers of individual mice were separated by 2-DE as described in the Materials and methods. The protein spots were quantified from colloidal Coomassie blue-stained gels using ImageMaster^TM^ 2D Elite software. Spot intensities were normalized to the total spot intensity for each gel and expressed as the mean percentage value ± SD (*n* = 4 for each group). Proteins that were significantly different (Student's *t*-test; *p* < 0.05) between the wild type and Nrf2 null mice are shown.

**Table 7:** Proteins identified as Nrf2 dependent in two or more analyses.

SwissProt acc. no.	Protein name	iTRAQ Analysis 1		iTRAQ Analysis 2		2DE gel analysis	
Fold-change	*p*	Fold-change	*p*	Fold-change	*p*
Q8VCW8	Acyl-CoA synthetase family member 2, mitochondrial	0.83	0.030	0.86	0.132		
P31786	Acyl-CoA-binding protein	1.26	0.073	1.18	0.207		
Q91VA0	Acyl-coenzyme A synthetase ACSM1, mitochondrial	0.78	0.039	0.81	0.001		
Q9JII6	Alcohol dehydrogenase [NADP+]	0.90	0.121	0.85	0.003		
P24549	Aldehyde dehydrogenase family 1, subfamily A1	0.76	0.127	0.72	0.021	0.78	0.003
P16460	Argininosuccinate synthase	1.26	0.162	1.25	0.038		
Q91X77	Cytochrome P450 2C50	0.65	0.162	0.58	0.001		
Q05816	Fatty acid-binding protein, epidermal	2.97	0.009	1.79	0.005		
Q9QXD6	Fructose-1,6-bisphosphatase 1	1.14	0.144	1.18	0.117		
P30115	Glutathione S-transferase, alpha 3			0.70	0.001	0.80	0.010
P10649	Glutathione S-transferase, mu 1	0.42	0.009	0.44	0.001	0.60	0.016
P19157	Glutathione S-transferase, pi 1	0.55	0.011	0.62	0.001	0.55	0.009
Q8VCC2	Liver carboxylesterase 1	0.58	0.042	0.56	0.001		
P06151	L-lactate dehydrogenase A chain	1.18	0.178	1.20	0.086		
Q8VBT2	L-serine dehydratase	1.51	0.078	1.34	0.183		
P02762	Major urinary protein 6	0.35	0.057			0.36	0.001
Q99P30	Peroxisomal coenzyme A diphosphatase NUDT7	1.43	0.044	1.13	0.039		
Q61207	Sulfated glycoprotein 1	1.22	0.121	1.21	0.057		
O70475	UDP-glucose 6-dehydrogenase	0.81	0.061	0.73	0.183		
P17717	UDP-glucuronosyltransferase 2B5	0.55	0.004	0.55	0.001		

Each protein was significantly (*p* < 0.05, Student *t*-test) over- or underexpressed in Nrf2^(−/−)^ mice compared with the wild type controls in at least two out of the three independent. Fold changes are the ratios of the mean expression changes from 4 to 6 mice.

**Table 8:** Promoter analysis for the mouse genes encoding Nrf2-regulated proteins.

			String search (*dna*-*pattern*)	Matrix analysis (*patser*)				Highest scoring ARE		
SwissProt acc. no.	Protein name	Fold-change	Number of consensus sequences (RTGABNNNGCA)	Number of matching sequences	Highest score	Mean score	SD	Location from to		Sequence
P02762	Major urinary protein 6	0.35	0	14	4.89	2.03	1.07	−1935	−1923	ttccCTGTCACTAAGCAtgtt
P10649	Glutathione S-transferase Mu 1	0.41	4	15	4.40	2.42	1.09	−56	−44	gtggGCAGGACAAAACAgcgg
P19157	Glutathione S-transferase P 1	0.54	0	13	4.02	2.11	0.98	−68	−56	aacgTGTTGAGTCAGCAtccg
Q91WG8	Bifunctional UDP-N-acetylglucosamine 2-epimerase/N-acetylmannosamine kinase	0.55	0	12	5.95	2.50	1.70	−387	−375	gcagGGGTGGCAAAGCTtaaa
P17717	UDP-glucuronosyltransferase 2B5	0.55	1	13	5.59	2.40	1.23	−398	−386	cagtCCATGACTGAGTTtgaa
Q99P30	Peroxisomal coenzyme A diphosphatase NUDT7	1.41	1	8	4.68	2.49	1.14	−848	−836	caagGCATTACACAGCCcagg
Q8JZR0	Long-chain-fatty-acid-CoA ligase 5	1.57	1	10	7.66	2.56	1.90	−1234	−1222	cttaGAATGACCCAGCCcttg
Q91V92	ATP-citrate synthase	1.75	1	9	10.02	3.26	2.58	−1899	−1887	agaaAAATGACTAAGCAggta
Q8VCH0	3-ketoacyl-CoA thiolase B, peroxisomal	2.21	2	15	5.84	2.55	1.44	−137	−125	tgggGGAAGACTCAGGAagag
Q05816	Fatty acid-binding protein, epidermal	2.81	0	15	4.37	2.59	0.86	−1728	−1716	agtgGGATGTCGCAGCTcagg
Mean values for all Nrf2-regulated proteins		1.26	1.25	13.69	5.62	2.50	1.33			
Mean values for all down-regulated Nrf2-dependent proteins		0.57	1.00	15.40	5.20	2.54	1.23			
Mean values for all up-regulated Nrf2-dependent proteins		1.57	1.36	12.91	5.81	2.49	1.37			
Mean values for all proteins identified			1.21	13.20	6.48	2.03	1.62			

Sequences of the genes of Nrf2-regulated proteins were obtained from the ENSMBL mouse genome database and interrogated for ARE and ARE/like consensus sequences using the RSAT analysis software (http://rsat.ulb.ac.be/rsat/). Both string-based (*dna-pattern*) and matrix-based (*patser*) pattern searching strategies were adopted (see text for details). For the *dna-pattern* analysis, returned sequences were rated against the ‘perfect’ consensus sequence **RTGAB**NNN**GCA**. For the *patser* analysis, the number of sequences matching the position specific scoring matrix with a score > 1 are given, along with the highest score attained. For comparison, equivalent data from the entire set of identified proteins is included at the foot of the table.

## Appendix A Supplementary data

Supplementary Table 1 Promoter analysis for the mouse genes encoding Nrf2-regulated proteins. Sequences of the genes of Nrf2-regulated proteins were obtained from the ENSMBL mouse genome database and interrogated for ARE and ARE/like consensus sequences using the RSAT analysis software (http://rsat.ulb.ac.be/rsat/). Both matrix-based (*patser*) and string-based (*dna**-**pattern*) pattern searching strategies were adopted (see text for details). For the patser analysis, the number of sequences matching the position specific scoring matrix with a score > 1 are given, along with the highest score attained. For the dna-pattern analysis, returned sequences were rated against the ‘perfect’ consensus sequence RTGABNNNGCA. For comparison, equivalent data from the entire set of identified proteins is included at the foot of the table.
