# Supplementary material for: Proteomic analysis of Nrf2 deficient transgenic mice reveals cellular defence and lipid metabolism as primary Nrf2-dependent pathways in the liver
Source: J Proteomics. 2010 Jun 16;73(8):1612–31. doi: 10.1016/j.jprot.2010.03.018 (PMC2891861; doi:10.1016/j.jprot.2010.03.018)
Supplement: Supplementary Table 1 — Promoter analysis for the mouse genes encoding Nrf2-regulated proteins. Sequences of the genes of Nrf2-regulated proteins were obtained from the ENSMBL mouse genome database and interrogated for ARE and ARE/like consensus sequences using the RSAT analysis software (http://rsat.ulb.ac.be/rsat/). Both matrix-based (patser) and string-based (dna-pattern) pattern searching strategies were adopted (see text for details). For the patser analysis, the number of sequences matching the position specific scoring matrix with a score > 1 are given, along with the highest score attained. For the dna-pattern analysis, returned sequences were rated against the ‘perfect’ consensus sequence RTGABNNNGCA. For comparison, equivalent data from the entire set of identified proteins is included at the foot of the table. [file mmc1.doc]

**Supplementary table 1:** ***Promoter analysis for the mouse genes encoding Nrf2-regulated proteins.***  Sequences of the genes of Nrf2-regulated proteins were obtained from the ENSMBL mouse genome database and interrogated for ARE and ARE/like consensus sequences using the RSAT analysis software (<http://rsat.ulb.ac.be/rsat/>). Both matrix-based (*patser*) and string-based (*dna-pattern*) pattern searching strategies were adopted (see text for details). For the patser analysis, the number of sequences matching the position specific scoring matrix with a score >1 are given, along with the highest score attained. For the dna-pattern analysis, returned sequences were rated against the ‘perfect’ consensus sequence **RTGAB**NNN**GCA**. For comparison, equivalent data from the entire set of identified proteins is included at the foot of the table.

|  |  |  | Matrix analysis (*patser*) | | | |  | String search  (*dna-pattern*) |
| --- | --- | --- | --- | --- | --- | --- | --- | --- |
| SwissProt accession number | Protein name | Fold-change | Number of matching sequences | Highest score | Mean score | SD |  | Number of consensus sequences (RGABNNNGC) |
| P02762 | Major urinary protein 6 | 0.35 | 14 | 4.89 | 2.03 | 1.07 |  | 0 |
| P10649 | Glutathione S-transferase Mu 1 | 0.41 | 15 | 4.40 | 2.42 | 1.09 |  | 4 |
| Q61656 | Probable ATP-dependent RNA helicase DDX5 | 0.52 | 14 | 5.54 | 3.21 | 1.49 |  | 2 |
| Q63836 | Selenium-binding protein 2 | 0.52 | 11 | 5.20 | 2.19 | 1.16 |  | 0 |
| P19157 | Glutathione S-transferase P 1 | 0.54 | 13 | 4.02 | 2.11 | 0.98 |  | 0 |
| Q91WG8 | Bifunctional UDP-N-acetylglucosamine 2-epimerase | 0.55 | 12 | 5.95 | 2.50 | 1.70 |  | 0 |
| P17717 | UDP-glucuronosyltransferase 2B5 | 0.55 | 13 | 5.59 | 2.40 | 1.23 |  | 0 |
| Q8VCC2 | Liver carboxylesterase 1 | 0.57 | 11 | 5.53 | 2.91 | 1.65 |  | 3 |
| Q60991 | Cytochrome P450 7B1 | 0.57 | 10 | 10.95 | 4.34 | 2.96 |  | 1 |
| P46425 | Glutathione S-transferase P 2 | 0.58 | 18 | 6.32 | 2.79 | 1.46 |  | 1 |
| O35660 | Glutathione S-transferase Mu 6 | 0.62 | 23 | 7.94 | 3.66 | 1.96 |  | 3 |
| P24472 | Glutathione S-transferase A4 | 0.62 | 13 | 6.43 | 2.56 | 1.69 |  | 2 |
| Q91X77 | Cytochrome P450 2C50 | 0.63 | 7 | 8.62 | 3.97 | 2.82 |  | 3 |
| P00186 | Cytochrome P450 1A2 | 0.64 | 11 | 4.46 | 2.35 | 1.03 |  | 3 |
| P15105 | Glutamine synthetase | 0.69 | 14 | 3.98 | 2.41 | 1.07 |  | 0 |
| Q9JIF7 | Coatomer subunit beta | 0.72 | 5 | 3.44 | 2.37 | 0.82 |  | 3 |
| Q6XVG2 | Cytochrome P450 2C54 | 0.74 | 19 | 6.61 | 2.10 | 1.19 |  | 1 |
| O35490 | Betaine--homocysteine S-methyltransferase 1 | 0.75 | 20 | 7.99 | 2.43 | 1.70 |  | 3 |
| P24549 | Retinal dehydrogenase 1 | 0.75 | 14 | 8.46 | 2.98 | 2.06 |  | 1 |
| Q9Z2W0 | Aspartyl aminopeptidase | 0.76 | 9 | 7.24 | 3.49 | 1.67 |  | 0 |
| Q91XE8 | Transmembrane protein 205 | 0.78 | 24 | 5.07 | 2.01 | 0.91 |  | 2 |
| Q91VA0 | Acyl-coenzyme A synthetase ACSM1, mitochondrial | 0.79 | 24 | 6.03 | 3.27 | 1.16 |  | 1 |
| Q99J99 | 3-mercaptopyruvate sulfurtransferase | 0.79 | 13 | 6.95 | 3.00 | 1.63 |  | 2 |
| Q8CG76 | Aflatoxin B1 aldehyde reductase member 2 | 0.81 | 10 | 9.72 | 3.35 | 2.44 |  | 3 |
| O70475 | UDP-glucose 6-dehydrogenase | 0.81 | 15 | 10.48 | 2.97 | 2.43 |  | 0 |
| Q8R1G2 | Carboxymethylenebutenolidase homolog | 0.82 | 11 | 4.62 | 3.04 | 1.13 |  | 2 |
| Q91VR5 | ATP-dependent RNA helicase DDX1 | 0.83 | 11 | 4.49 | 2.58 | 1.01 |  | 2 |
| P70441 | Na(+)/H(+) exchange regulatory cofactor NHE-RF1 | 0.84 | 9 | 6.54 | 2.58 | 1.67 |  | 2 |
| Q8VCW8 | Acyl-CoA synthetase family member 2, mitochondrial | 0.84 | 14 | 5.62 | 2.58 | 1.42 |  | 0 |
| P62843 | 40S ribosomal protein S15 | 0.84 | 14 | 3.89 | 2.53 | 0.88 |  | 2 |
| Q8VCU1 | Liver carboxylesterase 31-like | 0.84 | 9 | 7.76 | 2.93 | 2.06 |  | 1 |
| P07759 | Serine protease inhibitor A3K | 0.85 | 21 | 5.51 | 2.49 | 1.55 |  | 2 |
| Q9D6Y7 | Peptide methionine sulfoxide reductase | 0.87 | 15 | 7.47 | 2.44 | 1.47 |  | 0 |
| P60867 | 40S ribosomal protein S20 | 0.88 | 17 | 7.94 | 3.45 | 2.07 |  | 1 |
| P11352 | Glutathione peroxidase 1 | 0.88 | 14 | 4.12 | 2.40 | 0.89 |  | 1 |
| P99029 | Peroxiredoxin-5, mitochondrial | 0.90 | 16 | 6.75 | 3.24 | 1.86 |  | 1 |
| P62908 | 40S ribosomal protein S3 | 0.90 | 16 | 6.34 | 3.14 | 1.78 |  | 0 |
| Q9QYG0 | Protein NDRG2 | 0.91 | 13 | 8.44 | 2.69 | 2.23 |  | 1 |
| Q9JII6 | Alcohol dehydrogenase [NADP+] | 0.92 | 11 | 3.37 | 2.20 | 0.86 |  | 0 |
| Q8BH00 | Aldehyde dehydrogenase family 8 member A1 | 1.07 | 16 | 5.68 | 2.52 | 1.22 |  | 1 |
| Q9DBJ1 | Phosphoglycerate mutase 1 | 1.07 | 8 | 7.99 | 2.77 | 2.25 |  | 0 |
| Q8QZR5 | Alanine aminotransferase 1 | 1.07 | 19 | 4.73 | 2.61 | 1.04 |  | 0 |
| P62897 | Cytochrome c, somatic | 1.08 | 9 | 4.01 | 2.13 | 0.96 |  | 0 |
| Q8BVI4 | Dihydropteridine reductase | 1.08 | 14 | 9.20 | 3.83 | 2.60 |  | 1 |
| Q9JHI5 | Isovaleryl-CoA dehydrogenase, mitochondrial | 1.09 | 16 | 7.47 | 3.68 | 2.17 |  | 3 |
| Q9D0F9 | Phosphoglucomutase-1 | 1.10 | 9 | 6.18 | 2.33 | 1.58 |  | 0 |
| P62991 | Ubiquitin | 1.10 | 20 | 12.79 | 3.69 | 3.29 |  | 2 |
| P45952 | Medium-chain specific acyl-CoA dehydrogenase | 1.10 | 11 | 5.59 | 2.70 | 1.55 |  | 2 |
| P97807 | Fumarate hydratase, mitochondrial | 1.11 | 9 | 5.91 | 3.47 | 1.84 |  | 3 |
| Q99J08 | SEC14-like protein 2 | 1.11 | 10 | 5.38 | 2.90 | 1.17 |  | 0 |
| O88569 | Heterogeneous nuclear ribonucleoproteins A2/B1 | 1.14 | 9 | 6.50 | 3.07 | 1.67 |  | 0 |
| Q9JHW2 | Nitrilase homolog 2 | 1.14 | 13 | 7.46 | 3.32 | 1.65 |  | 0 |
| P08226 | Apolipoprotein E | 1.14 | 17 | 6.19 | 2.88 | 1.58 |  | 2 |
| O88451 | Retinol dehydrogenase 7 | 1.14 | 14 | 6.39 | 2.55 | 1.41 |  | 0 |
| P00329 | Alcohol dehydrogenase 1 | 1.14 | 15 | 5.39 | 2.47 | 1.40 |  | 1 |
| P17742 | Peptidyl-prolyl cis-trans isomerase A | 1.15 | 10 | 7.99 | 3.54 | 2.28 |  | 3 |
| Q8BH95 | Enoyl-CoA hydratase, mitochondrial | 1.15 | 8 | 9.81 | 3.46 | 2.77 |  | 2 |
| Q8VCR2 | 17-beta hydroxysteroid dehydrogenase 13 | 1.16 | 11 | 7.21 | 2.18 | 1.74 |  | 0 |
| Q9QXD6 | Fructose-1,6-bisphosphatase 1 | 1.16 | 13 | 4.55 | 2.06 | 1.03 |  | 3 |
| P51660 | Peroxisomal multifunctional enzyme type 2 | 1.17 | 7 | 6.52 | 3.17 | 2.08 |  | 0 |
| Q9R0Q7 | Prostaglandin E synthase 3 | 1.17 | 9 | 3.25 | 2.25 | 0.77 |  | 1 |
| Q9JI75 | Ribosyldihydronicotinamide dehydrogenase | 1.18 | 4 | 5.16 | 3.14 | 2.10 |  | 1 |
| Q64105 | Sepiapterin reductase | 1.18 | 16 | 6.59 | 2.90 | 1.29 |  | 2 |
| Q8VCR7 | Abhydrolase domain-containing protein 14B | 1.19 | 13 | 6.08 | 2.74 | 1.43 |  | 0 |
| P00405 | Cytochrome c oxidase subunit 2 | 1.19 | 13 | 5.35 | 2.67 | 1.39 |  | 0 |
| Q8CHR6 | Dihydropyrimidine dehydrogenase [NADP+] | 1.20 | 12 | 3.07 | 2.10 | 0.57 |  | 2 |
| Q8VC12 | Probable urocanate hydratase | 1.20 | 10 | 6.09 | 2.73 | 1.50 |  | 1 |
| P06151 | L-lactate dehydrogenase A chain | 1.21 | 12 | 6.33 | 3.14 | 1.71 |  | 1 |
| P54775 | 26S protease regulatory subunit 6B | 1.21 | 11 | 5.05 | 2.89 | 1.30 |  | 1 |
| P24270 | Catalase | 1.21 | 12 | 4.23 | 2.29 | 0.94 |  | 2 |
| P50172 | Corticosteroid 11-beta-dehydrogenase isozyme 1 | 1.22 | 19 | 8.46 | 3.54 | 2.08 |  | 2 |
| P80316 | T-complex protein 1 subunit epsilon | 1.23 | 18 | 7.07 | 2.78 | 1.98 |  | 1 |
| P32020 | Non-specific lipid-transfer protein | 1.23 | 10 | 6.60 | 2.78 | 1.87 |  | 0 |
| Q61207 | Sulfated glycoprotein 1 | 1.23 | 10 | 9.36 | 4.05 | 2.82 |  | 1 |
| Q60932 | Voltage-dependent anion-selective channel protein 1 | 1.24 | 17 | 10.14 | 3.38 | 2.83 |  | 0 |
| P84078 | ADP-ribosylation factor 1 | 1.24 | 13 | 6.57 | 2.54 | 1.43 |  | 1 |
| P55096 | ATP-binding cassette sub-family D member 3 | 1.25 | 7 | 7.99 | 3.04 | 2.40 |  | 4 |
| Q9Z2I8 | Succinyl-CoA ligase [GDP-forming] subunit beta | 1.25 | 13 | 7.34 | 2.77 | 1.90 |  | 2 |
| Q9DD20 | Methyltransferase-like protein 7B | 1.25 | 14 | 9.12 | 2.87 | 2.10 |  | 0 |
| Q9QXF8 | Glycine N-methyltransferase | 1.25 | 15 | 10.23 | 3.76 | 2.62 |  | 0 |
| P19096 | Fatty acid synthase | 1.27 | 21 | 5.92 | 2.62 | 1.22 |  | 2 |
| Q99PG0 | Arylacetamide deacetylase | 1.28 | 14 | 4.75 | 2.10 | 1.07 |  | 0 |
| P12787 | Cytochrome c oxidase subunit 5A, mitochondrial | 1.29 | 17 | 9.32 | 2.92 | 2.09 |  | 3 |
| Q9QXE0 | 2-hydroxyacyl-CoA lyase 1 | 1.30 | 13 | 9.16 | 3.44 | 2.35 |  | 1 |
| P27659 | 60S ribosomal protein L3 | 1.30 | 11 | 7.99 | 2.91 | 1.93 |  | 2 |
| P16460 | Argininosuccinate synthase | 1.30 | 15 | 7.99 | 2.76 | 1.77 |  | 0 |
| Q9R0H0 | Peroxisomal acyl-coenzyme A oxidase 1 | 1.31 | 11 | 7.06 | 2.07 | 1.75 |  | 2 |
| P35492 | Histidine ammonia-lyase | 1.31 | 13 | 4.33 | 2.44 | 1.05 |  | 3 |
| P18242 | Cathepsin D | 1.31 | 13 | 8.49 | 3.97 | 2.28 |  | 1 |
| P31786 | Acyl-CoA-binding protein | 1.32 | 13 | 5.13 | 2.81 | 1.18 |  | 0 |
| Q64459 | Cytochrome P450 3A11 | 1.32 | 7 | 6.11 | 2.21 | 1.84 |  | 0 |
| P17665 | Cytochrome c oxidase subunit 7C, mitochondrial | 1.33 | 7 | 5.19 | 2.42 | 1.42 |  | 0 |
| P05201 | Aspartate aminotransferase, cytoplasmic | 1.34 | 11 | 5.86 | 3.23 | 1.40 |  | 2 |
| P25688 | Uricase | 1.38 | 16 | 3.83 | 1.92 | 0.70 |  | 0 |
| Q9QXD1 | Peroxisomal acyl-coenzyme A oxidase 2 | 1.41 | 15 | 12.05 | 3.67 | 3.09 |  | 3 |
| Q99P30 | Peroxisomal coenzyme A diphosphatase NUDT7 | 1.41 | 8 | 4.68 | 2.49 | 1.14 |  | 1 |
| P20108 | Thioredoxin-dependent peroxide reductase | 1.41 | 6 | 6.86 | 3.55 | 1.92 |  | 2 |
| Q8VCX1 | 3-oxo-5-beta-steroid 4-dehydrogenase | 1.42 | 15 | 6.31 | 2.72 | 1.74 |  | 1 |
| Q9DBM2 | Peroxisomal bifunctional enzyme | 1.48 | 13 | 8.68 | 3.29 | 2.09 |  | 2 |
| Q8JZR0 | Long-chain-fatty-acid--CoA ligase 5 | 1.57 | 10 | 7.66 | 2.56 | 1.90 |  | 1 |
| Q8VBT2 | L-serine dehydratase | 1.63 | 10 | 4.60 | 2.46 | 1.10 |  | 2 |
| Q91V92 | ATP-citrate synthase | 1.75 | 9 | 10.02 | 3.26 | 2.58 |  | 1 |
| P13516 | Acyl-CoA desaturase 1 | 2.18 | 17 | 5.78 | 2.44 | 1.45 |  | 1 |
| Q8VCH0 | 3-ketoacyl-CoA thiolase B, peroxisomal | 2.21 | 15 | 5.84 | 2.55 | 1.44 |  | 2 |
| Q05816 | Fatty acid-binding protein, epidermal | 2.81 | 15 | 4.37 | 2.59 | 0.86 |  | 0 |
|  | **Mean values for all up- or down-regulated proteins in Nrf2 null mice** | **1.08** | **12.99** | **6.57** | **2.83** | **1.66** |  | **1.24** |
|  | **Mean values for all proteins down-regulated in Nrf2 null mice** | **0.63** | **14.22** | **6.16** | **2.76** | **1.50** |  | **1.52** |
|  | **Mean values for all proteins up-regulated in Nrf2 null mice** | **1.40** | **12.70** | **6.89** | **2.85** | **1.74** |  | **1.25** |
|  | **Mean values for all proteins identified** |  |  | 6.48 | 2.80 | 1.62 |  | **1.23** |
